# Supplementary material for: Association of thoracic spine deformity and cardiovascular disease in a mouse model for Marfan syndrome
Source: PLoS One. 2019 Nov 14;14(11):e0224581. doi: 10.1371/journal.pone.0224581 (PMC6855660; doi:10.1371/journal.pone.0224581)
Supplement: S1 Data Set — (DOC) [file pone.0224581.s001.doc]

**Supporting Information – Individual measurements used to build graphs and perform statistical analysis.**

**Area of aorta’s lumen**

| WT | MFS^-^ | MFS^+^ |
| --- | --- | --- |
| 61203.350000 | 47989.420000 | 3236.002000 |
| 75514.500000 | 53219.380000 | 3243.799000 |
| 60712.960000 | 56780.320000 | 3890.889000 |
| 75516.590000 | 56898.070000 | 4064.823000 |
| 64607.030000 | 64796.710000 | 4122.351000 |
| 57471.030000 | 16782.860000 | 4556.621000 |
| 58991.270000 | 34448.590000 | 4714.695000 |
| 60768.710000 | 39509.730000 | 4714.695000 |
| 57709.930000 | 45479.880000 | 9039.892000 |
| 58813.860000 | 45572.230000 | 12630.950000 |
|  | 51981.850000 | 12630.950000 |
|  | 52042.200000 | 18770.800000 |
|  | 56981.950000 | 18770.800000 |
|  | 57885.570000 | 24224.050000 |
|  | 58529.390000 | 26186.160000 |
|  | 59091.800000 | 28274.700000 |
|  | 61154.520000 | 28355.790000 |
|  | 64895.410000 | 28355.790000 |
|  | 65538.660000 | 34970.010000 |
|  | 66407.880000 | 34970.010000 |
|  | 68708.390000 | 38016.650000 |
|  | 39575.610000 | 41421.160000 |
|  | 70406.860000 | 41563.420000 |
|  | 81431.450000 | 41903.170000 |
|  |  | 44465.270000 |
|  |  | 45473.030000 |
|  |  | 45473.030000 |
|  |  | 46689.300000 |
|  |  | 46689.300000 |
|  |  | 47119.860000 |
|  |  | 47119.860000 |
|  |  | 48755.200000 |
|  |  | 48755.200000 |
|  |  | 51585.690000 |
|  |  | 52117.610000 |
|  |  | 52117.610000 |
|  |  | 52651.590000 |
|  |  | 53314.340000 |
|  |  | 55525.840000 |
|  |  | 56512.860000 |
|  |  | 57640.610000 |
|  |  | 58836.390000 |
|  |  | 58978.160000 |
|  |  | 58978.160000 |
|  |  | 60892.710000 |
|  |  | 62120.240000 |
|  |  | 62151.250000 |
|  |  | 62550.520000 |
|  |  | 63423.340000 |
|  |  | 63631.070000 |
|  |  | 65992.470000 |
|  |  | 66614.730000 |
|  |  | 69251.880000 |
|  |  | 70421.310000 |
|  |  | 70743.430000 |
|  |  | 71764.620000 |
|  |  | 72373.750000 |
|  |  | 73478.350000 |
|  |  | 73905.880000 |

**Thickness of elastic fibers**

| WT | MFS^v-^ | MFS^v+^ |
| --- | --- | --- |
| 1.276 | 1.4460 | 1.0280 |
| 1.838 | 2.5530 | 1.5690 |
| 1.224 | 1.4560 | 1.6260 |
| 1.138 | 1.3610 | 1.8080 |
| 1.838 | 1.2690 | 0.6020 |
| 1.582 | 1.4080 | 1.2760 |
| 1.282 | 0.6860 | 0.7320 |
| 1.757 | 2.7000 | 0.5710 |
| 1.138 | 2.0300 | 0.8760 |
| 1.248 | 1.4460 | 1.2030 |
| 1.214 | 1.0280 | 1.6780 |
| 1.408 | 2.0440 | 0.8420 |
| 1.641 | 1.5620 | 1.7700 |
| 1.286 | 0.9960 | 1.8410 |
| 1.395 | 1.3720 | 1.4490 |
| 1.644 | 0.9510 | 0.6420 |
| 1.744 | 1.0520 | 0.5320 |
| 1.636 | 1.3290 | 0.7010 |
| 1.298 | 1.6260 | 0.7970 |
| 1.410 | 0.8680 | 0.8660 |
| 1.646 | 0.9400 | 0.6700 |
| 1.473 | 1.7350 | 0.6630 |
| 1.706 | 0.9510 | 1.0230 |
| 1.493 | 1.1570 | 0.8280 |
| 1.867 | 1.5990 | 0.8170 |
| 1.298 | 1.5220 | 1.0210 |
| 1.302 | 1.3570 | 0.8720 |
| 1.636 | 1.0760 | 1.2060 |
| 1.209 | 0.7320 | 0.7360 |
| 1.442 | 1.3320 | 0.8850 |
| 1.622 | 1.2880 | 1.2660 |
| 1.422 | 0.7840 | 0.6460 |
| 1.149 | 1.7950 | 0.4910 |
| 1.576 | 1.0830 | 1.1000 |
| 1.437 | 1.1820 | 1.1550 |
| 1.493 | 0.5710 | 0.7110 |
| 1.622 | 1.5340 | 0.7490 |
| 1.511 | 1.1940 | 0.7520 |
| 1.672 | 1.3640 | 1.4970 |
| 1.547 | 1.1190 | 1.2950 |
| 1.343 | 1.3750 | 0.9500 |
| 1.260 | 1.1190 | 0.7000 |
| 1.354 | 1.6170 | 1.1240 |
| 1.239 | 1.4690 | 0.4360 |
| 1.493 | 1.1090 | 1.0330 |
| 1.644 | 1.4610 | 0.6480 |
| 1.560 | 0.8280 | 0.9910 |
| 1.211 | 1.5300 | 0.9820 |
| 1.321 | 1.1410 | 0.5260 |
| 1.065 | 1.2550 | 0.6940 |
| 1.273 | 1.5070 | 1.0760 |
| 1.333 | 1.4930 | 1.0120 |
| 1.053 | 1.1230 | 0.8200 |
| 1.669 | 0.8310 | 0.7360 |
| 1.329 | 1.1410 | 0.9130 |
| 1.052 | 1.4930 | 0.7610 |
| 1.298 | 1.1330 | 0.9160 |
| 1.128 | 0.9670 | 1.2060 |
| 1.387 | 1.0210 | 0.8870 |
| 1.211 | 1.4360 | 1.5890 |
| 1.211 | 1.5430 | 1.1590 |
| 1.514 | 1.4940 | 1.2250 |
| 1.841 | 0.8610 | 0.6840 |
| 1.734 | 0.9460 | 0.7610 |
| 1.081 | 1.7150 | 1.4290 |
| 1.557 | 1.5700 | 0.8870 |
| 1.511 | 1.6200 | 0.6020 |
| 1.427 | 1.5490 | 1.1090 |
| 1.664 | 1.8570 | 1.6810 |
| 1.070 | 1.0690 | 1.2760 |
| 1.590 | 2.1930 | 0.9400 |
| 1.671 | 1.4540 | 0.5960 |
| 1.833 | 1.7770 | 1.2790 |
| 1.700 | 0.8880 | 1.0250 |
| 1.233 | 1.0390 | 1.1060 |
| 1.502 | 0.9920 | 0.5710 |
| 1.622 | 1.2650 | 1.5320 |
| 1.248 | 1.3320 | 0.7700 |
| 1.161 | 1.3750 | 0.7660 |
| 1.056 | 1.4460 | 0.5900 |
| 1.161 | 1.3320 | 1.8720 |
| 1.230 | 1.3290 | 1.0250 |
| 2.045 | 1.6190 | 1.1940 |
| 1.411 | 0.8420 | 0.8550 |
| 1.803 | 1.0280 | 1.4560 |
| 2.059 | 0.8510 | 1.0350 |
| 1.935 | 0.9920 | 0.7700 |
| 1.628 | 1.0760 | 1.3750 |
| 1.720 | 0.9510 | 0.7320 |
| 2.326 | 0.9700 | 1.1000 |
| 2.009 | 1.4080 | 0.8510 |
| 1.720 | 0.8380 | 1.0210 |
| 1.710 | 1.0210 | 0.5960 |
| 1.020 | 0.7270 | 1.4490 |
| 1.680 | 1.1090 | 1.3640 |
| 2.000 | 0.9200 | 1.3950 |
| 1.622 | 0.9510 | 0.9200 |
| 1.608 | 0.9510 | 1.4080 |
| 1.451 | 1.9570 | 1.0660 |
| 1.443 | 1.1940 | 0.6860 |
| 2.100 | 1.6170 | 0.6420 |
| 1.189 | 1.1060 | 1.0120 |
| 1.760 | 1.1060 | 1.3090 |
| 1.298 | 1.1820 | 0.8530 |
| 1.410 | 1.7950 | 0.8660 |
| 1.628 | 1.8830 | 0.9480 |
| 1.803 | 1.6160 | 1.1330 |
| 1.511 | 1.5410 | 0.9670 |
| 1.628 | 0.8550 | 0.8200 |
| 1.352 | 1.6170 | 1.0120 |
| 1.487 | 1.6170 | 1.8390 |
| 1.511 | 0.9700 | 1.4730 |
| 1.487 | 1.2090 | 0.9460 |
| 1.518 | 1.0250 | 1.1080 |
| 2.077 | 1.0350 | 0.8600 |
| 1.730 | 0.8880 | 1.1750 |
| 1.298 | 0.8880 | 1.1410 |
| 1.410 | 1.2180 | 1.0650 |
| 1.681 | 0.6190 | 0.7610 |
| 1.384 | 1.1910 | 1.3430 |
| 1.371 | 0.8680 | 1.7450 |
| 1.263 | 0.9360 | 1.1570 |
| 1.189 | 1.0250 | 0.7610 |
| 1.760 | 1.1190 | 0.9460 |
| 1.410 | 1.1910 | 0.9260 |
| 1.628 | 0.8510 | 1.0210 |
| 1.803 | 1.1450 | 0.4760 |
| 1.511 | 1.2650 | 1.0910 |
| 1.628 | 1.0390 | 1.2270 |
| 1.352 | 1.2960 | 1.7750 |
| 1.487 | 0.6860 | 1.0910 |
| 1.511 | 1.1570 | 1.0230 |
| 1.487 | 1.1570 | 0.9550 |
| 1.518 | 1.3850 | 1.1590 |
| 2.077 | 1.1450 | 1.2270 |
| 1.730 | 1.0280 | 1.2950 |
| 1.298 | 1.1570 | 1.2270 |
| 1.410 | 0.8880 | 0.9530 |
| 1.681 | 0.7700 | 1.3630 |
| 1.384 | 1.1570 | 0.8610 |
| 1.371 | 1.3450 | 0.9530 |
| 1.263 | 0.8550 | 0.9630 |
| 1.471 | 0.5710 | 1.1270 |
| 1.298 | 1.8680 | 0.8950 |
| 1.125 | 0.5710 | 0.9910 |
| 1.557 | 0.7270 | 0.8200 |
| 1.838 | 1.3610 | 0.6810 |
| 1.189 | 0.6860 | 0.8870 |
| 1.149 | 1.2790 | 1.1450 |
| 1.548 | 0.6860 | 1.6340 |
| 1.518 | 0.4250 | 1.0830 |
| 1.730 | 0.7270 | 1.1450 |
| 1.514 | 1.0350 | 0.9590 |
| 0.997 | 0.7700 | 0.9630 |
| 1.905 | 1.2180 | 0.7840 |
| 1.473 | 0.6860 | 1.0250 |
| 1.419 | 0.7700 | 1.5040 |
| 1.557 | 1.2180 | 1.9030 |
| 1.744 | 0.6860 | 0.8880 |
| 1.518 | 0.9400 | 0.8070 |
| 1.710 | 1.0830 | 0.8030 |
| 1.506 | 0.9920 | 0.6810 |
| 1.626 | 1.0390 | 0.5450 |
| 1.518 | 1.1450 | 1.0660 |
| 1.626 | 1.0250 | 1.1450 |
| 1.622 | 0.9700 | 1.6340 |
| 1.422 | 1.3720 | 1.0830 |
| 1.487 | 1.3450 | 1.1450 |
| 1.628 | 1.1450 | 0.9590 |
| 1.487 | 1.1350 | 0.9630 |
| 1.493 | 1.3750 | 0.7840 |
| 1.358 | 1.3020 | 1.0250 |
| 1.514 | 1.5620 | 1.5040 |
| 1.410 | 0.9920 | 1.9030 |
| 1.298 | 1.6780 | 0.8880 |
| 1.783 | 1.5530 | 0.8070 |
| 1.518 | 0.9400 | 0.8030 |
| 1.760 | 0.9400 | 0.6810 |
| 1.107 | 0.8550 | 0.5450 |
| 1.594 | 1.7040 | 1.0660 |
| 1.347 | 1.6170 | 1.1700 |
| 1.238 | 1.1940 | 1.1750 |
| 1.300 | 0.8880 | 1.1890 |
| 1.248 | 1.3120 | 1.2760 |
| 1.116 | 0.9510 | 0.8200 |
| 1.479 | 0.9510 | 0.6470 |
| 1.432 | 1.0350 | 1.4130 |
| 1.408 | 1.1570 | 1.3680 |
| 1.320 | 1.3720 | 1.2840 |
| 1.825 | 0.8510 | 0.8280 |
| 1.921 | 1.2650 | 0.8170 |
| 2.078 | 0.6020 | 1.2270 |
| 1.511 | 0.9920 | 0.9130 |
| 1.783 | 1.0660 | 1.2910 |
| 1.427 | 1.0660 | 1.1890 |
| 1.653 | 0.9960 | 0.8870 |
| 1.732 | 1.0280 | 0.9260 |
| 1.427 | 0.8030 | 1.0520 |
| 1.358 | 1.1850 | 1.3950 |
| 2.200 | 1.0350 | 0.2200 |
| 1.497 | 0.9700 | 1.2180 |
| 1.358 | 1.1090 | 1.3750 |
| 1.302 | 1.0250 | 1.6050 |
| 1.406 | 1.1060 | 1.1850 |
| 1.298 | 0.8510 | 0.8380 |
| 1.506 | 1.0360 | 1.2090 |
| 1.710 | 0.9630 | 1.3290 |
| 1.410 | 0.6480 | 1.3750 |
| 1.518 | 0.9510 | 0.7840 |
| 1.427 | 1.1450 | 0.8550 |
| 1.432 | 1.1450 | 1.5340 |
| 1.286 | 1.1570 | 1.4080 |
| 1.408 | 1.0390 | 1.7520 |
| 1.189 | 0.9920 | 1.0250 |
| 1.081 | 0.9700 | 1.6050 |
| 1.406 | 1.4080 | 1.3240 |
| 1.410 | 1.0520 | 0.7320 |
| 1.654 | 1.0350 | 0.9360 |
| 0.924 | 1.2650 | 1.2990 |
| 1.026 | 1.5340 | 0.9400 |
| 1.260 | 1.3450 | 1.0660 |
| 1.533 | 1.2650 | 0.9040 |
| 1.309 | 1.4080 | 1.3450 |
| 1.211 | 0.7220 | 1.2090 |
| 1.680 | 1.5340 | 0.9040 |
| 1.493 | 1.3450 | 0.8420 |
| 1.395 | 0.9510 | 1.1420 |
| 1.440 | 0.9360 | 1.3290 |
| 1.680 | 1.5950 | 1.1850 |
| 1.238 | 2.0650 | 1.0660 |
| 1.385 | 1.0530 | 1.1820 |
| 1.610 | 1.1500 | 0.7840 |
| 1.615 | 1.5810 | 0.8510 |
| 1.491 | 1.8080 | 0.6860 |
| 1.511 | 0.6700 | 1.3610 |
| 1.487 | 1.0640 | 1.5050 |
| 1.217 | 0.9570 | 1.4490 |
| 1.628 | 1.1310 | 0.8510 |
| 1.358 | 0.7740 | 0.7840 |
| 1.646 | 1.3160 | 0.8510 |
| 1.560 | 1.0950 | 0.8550 |
| 1.511 | 1.3620 | 0.8550 |
| 1.300 | 0.7520 | 1.1570 |
| 1.277 | 1.4000 | 0.7850 |
| 2.597 | 1.5080 | 1.0900 |
| 1.841 | 0.6480 | 0.9150 |
| 1.813 | 1.1090 | 0.9190 |
| 1.574 | 1.6050 | 1.0170 |
| 1.744 | 1.8330 | 0.7840 |
| 2.133 | 1.4490 | 0.8460 |
| 1.913 | 1.8270 | 1.2210 |
| 1.518 | 2.1010 | 1.1510 |
| 1.946 | 1.6370 | 1.1330 |
| 1.626 | 0.7610 | 0.8290 |
| 1.087 | 1.1090 | 0.7520 |
| 1.368 | 1.1910 | 0.9310 |
| 1.646 | 1.8030 | 1.4380 |
| 1.387 | 1.3210 | 1.0330 |
| 1.511 | 1.8680 | 1.0760 |
| 1.309 | 1.2450 | 1.0160 |
| 2.020 | 0.9810 | 0.7240 |
| 1.481 | 0.8770 | 0.7940 |
| 1.628 | 0.9630 | 1.1330 |
| 1.622 | 1.6090 | 1.0980 |
| 1.757 | 1.3450 | 1.1500 |
| 1.155 | 1.0640 | 0.9810 |
| 1.298 | 1.3830 | 0.6470 |
| 1.514 | 0.9810 | 0.6470 |
| 1.730 | 1.8110 | 0.8510 |
| 1.622 | 1.0690 | 1.5990 |
| 1.800 | 1.1700 | 0.7670 |
| 1.985 | 0.6470 | 1.1750 |
| 1.149 | 0.9630 | 0.7440 |
| 1.081 | 1.3850 | 0.9090 |
| 1.302 | 1.1450 | 0.8510 |
| 1.113 | 1.1090 | 1.7150 |
| 1.451 | 1.2650 | 1.1570 |
| 1.410 | 1.1090 | 1.0760 |
| 1.307 | 0.9400 | 1.1090 |
| 1.065 | 0.3830 | 1.0210 |
| 1.626 | 1.1850 | 1.3950 |
| 1.451 | 1.0520 | 1.0250 |
| 1.352 | 0.9160 | 1.7000 |
| 1.897 | 1.4260 | 2.2270 |
| 1.819 | 1.3720 | 1.3720 |
| 1.419 | 0.9400 | 1.3850 |
| 1.432 | 1.1570 | 0.5110 |
| 1.318 | 0.9700 | 1.1190 |
| 1.300 | 0.8680 | 0.9510 |
| 1.471 | 0.9510 | 1.2030 |
| 1.473 | 0.9879 | 1.2760 |
| 1.567 | 0.6650 | 1.3950 |
| 1.408 | 0.6860 | 0.7700 |
| 1.579 | 1.3020 | 1.1420 |
| 1.493 | 1.0250 | 1.0350 |
| 1.205 | 0.9920 | 1.7890 |
| 1.471 | 1.2030 | 1.0280 |
| 1.717 | 1.1190 | 0.9200 |
| 1.063 | 1.0250 | 1.1450 |
| 1.481 | 0.8680 | 1.7890 |
| 2.133 | 0.8800 | 1.6190 |
| 1.838 | 0.7840 | 1.2560 |
| 2.281 | 1.2180 | 0.9960 |
| 1.406 | 0.6810 | 1.0660 |
| 2.088 | 1.4490 | 1.0210 |
| 1.730 | 1.0830 | 1.1090 |
| 1.189 | 0.8510 | 1.5150 |
| 1.626 | 1.6370 | 1.3720 |
| 1.302 | 1.0520 | 1.0830 |
| 1.410 | 0.6020 | 0.9920 |
| 1.302 | 1.0520 | 1.1940 |
| 1.529 | 1.2760 | 1.1570 |
| 1.410 | 1.4460 | 1.2030 |
| 1.410 | 0.6200 | 0.6650 |
| 1.744 | 1.2760 | 1.7230 |
| 1.750 | 0.7700 | 1.3950 |
| 1.680 | 0.9400 | 1.8680 |
| 1.905 | 1.0520 | 1.2760 |
| 1.560 | 0.9400 | 1.0660 |
| 1.646 | 1.2990 | 1.2790 |
| 1.921 | 1.1090 | 1.3720 |
| 1.298 | 1.1060 | 1.2270 |
| 1.128 | 1.1190 | 0.8280 |
| 1.309 | 1.4690 | 1.2270 |
|  | 1.1090 | 1.0210 |
|  | 1.1190 | 1.3970 |
|  | 1.7460 | 1.0210 |
|  | 1.4460 | 0.7240 |
|  | 1.1890 | 1.2270 |
|  | 1.9120 | 1.0120 |
|  | 1.4400 | 0.7520 |
|  | 1.1910 | 0.8200 |
|  | 1.0760 | 1.1590 |
|  | 1.2650 | 1.3480 |
|  | 1.1570 | 1.0890 |
|  | 0.8420 | 1.0300 |
|  | 0.9700 | 1.3080 |
|  | 0.9040 | 0.9670 |
|  | 1.6260 | 1.2270 |
|  | 1.2090 | 0.8530 |
|  | 1.5080 | 0.9670 |
|  | 0.9510 | 0.8870 |
|  | 1.1060 | 0.9550 |
|  | 1.5710 | 0.7360 |
|  | 0.8850 | 1.2500 |
|  | 1.5520 | 1.1160 |
|  | 1.0390 | 0.9670 |
|  | 0.8850 | 0.7760 |
|  | 1.0410 | 1.2790 |
|  | 0.7760 | 0.9670 |
|  | 1.8090 | 0.9630 |
|  | 0.9630 | 1.1590 |
|  | 1.1000 | 1.0120 |
|  | 0.7760 | 0.9160 |
|  | 1.2270 | 1.2550 |
|  | 1.1080 | 0.8720 |
|  | 0.8530 | 1.3450 |
|  | 0.6420 | 1.1350 |
|  | 0.9480 | 1.2030 |
|  | 1.1270 | 0.9160 |
|  | 1.4550 | 1.4690 |
|  | 1.1270 | 1.0250 |
|  | 0.6810 | 0.8760 |
|  | 0.7520 | 1.0900 |
|  | 0.7490 | 1.2650 |
|  | 1.0210 | 1.3000 |
|  | 1.3000 | 1.1910 |
|  | 0.6130 | 0.8720 |
|  | 0.4810 | 1.9170 |
|  | 0.6810 | 1.3080 |
|  | 0.8280 | 0.9080 |
|  | 0.8310 | 1.2950 |
|  | 0.6810 | 1.0230 |
|  | 0.8280 | 0.7760 |
|  | 1.3510 | 0.8850 |
|  | 1.3080 | 0.8870 |
|  | 1.2270 | 1.2330 |
|  | 0.8870 | 1.3970 |
|  | 0.7490 | 0.9530 |
|  | 0.6840 | 1.0230 |
|  | 0.6420 | 0.7490 |
|  | 0.8200 | 1.1650 |
|  | 1.0630 | 1.3970 |
|  | 0.9820 | 0.7970 |
|  | 0.8200 | 1.0910 |
|  | 1.1080 | 0.7490 |
|  | 1.1330 | 0.8420 |
|  | 1.3610 | 0.9700 |
|  | 0.9920 | 1.1420 |
|  | 0.8030 | 1.3850 |
|  | 1.0520 | 1.1060 |
|  | 0.6860 | 0.8380 |
|  | 0.9510 | 1.1090 |
|  | 0.7610 | 0.9040 |
|  | 1.5640 | 1.1940 |
|  | 0.9950 | 1.2390 |
|  | 1.2180 | 0.9400 |
|  | 0.7840 | 0.5710 |
|  | 1.1820 | 1.0280 |
|  | 0.9700 | 0.8720 |
|  | 0.7660 | 1.3080 |
|  | 1.6520 | 0.9260 |
|  | 1.7020 | 2.2140 |
|  | 1.1450 | 0.4910 |
|  | 1.4460 | 0.4299 |
|  | 1.2270 | 0.5792 |
|  | 1.2550 | 0.9346 |
|  | 1.2660 | 0.3714 |
|  | 0.6810 | 0.6940 |
|  | 0.1710 | 0.7610 |
|  | 1.0050 | 0.9550 |
|  | 1.2120 | 1.1330 |
|  | 1.2120 | 1.1910 |
|  | 1.5110 | 1.0300 |
|  | 1.5110 | 0.7110 |
|  | 0.8220 | 0.8170 |
|  | 1.0760 | 0.7610 |
|  | 0.6810 | 0.7760 |
|  | 1.2510 | 1.3410 |
|  | 1.1160 | 1.2500 |
|  | 1.2270 | 0.7520 |
|  | 0.9750 | 0.6840 |
|  | 0.9750 | 1.5790 |
|  | 1.1590 | 0.7610 |
|  | 1.2840 | 1.2760 |
|  | 0.8720 | 1.4260 |
|  | 0.8660 | 0.8510 |
|  | 1.3610 | 1.1000 |
|  | 1.2090 | 1.8800 |
|  | 1.2650 | 1.3610 |
|  | 1.5640 | 1.3640 |
|  | 1.3020 | 0.9040 |
|  | 1.5640 | 1.0250 |
|  | 1.5220 | 1.3020 |
|  | 1.0830 | 0.8680 |
|  | 1.1190 | 0.6020 |
|  | 1.1420 | 1.0250 |
|  | 1.3850 | 1.4460 |
|  | 1.8370 | 0.9200 |
|  | 1.4880 | 1.3850 |
|  | 0.9510 | 1.3290 |
|  | 0.7220 | 1.2790 |
|  | 1.3540 | 1.2560 |
|  | 1.0030 | 1.0210 |
|  | 1.7150 | 0.7490 |
|  | 1.3620 | 0.7760 |
|  | 1.2130 | 1.2060 |
|  | 1.3830 | 0.8310 |
|  | 1.7180 | 1.5950 |
|  | 0.7670 | 1.1080 |
|  | 1.5810 | 0.9080 |
|  | 0.9090 | 1.3410 |
|  | 1.2850 | 1.0410 |
|  | 0.9090 | 0.8870 |
|  | 1.1500 | 0.8170 |
|  | 1.2450 | 1.0230 |
|  | 1.2760 | 1.0760 |
|  | 1.7310 | 1.3630 |
|  | 1.2130 | 1.0300 |
|  | 1.6230 | 1.3630 |
|  | 0.6470 | 0.7110 |
|  | 1.2850 | 1.2790 |
|  | 0.9090 | 1.2930 |
|  | 1.2450 | 0.9750 |
|  | 1.2760 | 0.9530 |
|  | 1.1500 | 0.9670 |
|  | 1.7310 | 1.0390 |
|  | 1.2130 | 1.3000 |
|  | 1.6230 | 0.7520 |
|  | 1.1500 | 1.2720 |
|  | 1.4470 | 0.9630 |
|  | 1.6200 | 1.4310 |
|  | 1.5340 | 0.8720 |
|  | 1.2450 | 0.9630 |
|  | 1.2030 | 1.4490 |
|  | 0.6470 | 1.2180 |
|  | 1.8030 | 0.8380 |
|  | 0.9700 | 0.8380 |
|  | 1.2180 | 1.1420 |
|  | 1.2790 | 1.2270 |
|  | 1.2090 | 0.8550 |
|  | 1.0390 | 1.2790 |
|  | 1.3020 | 0.9630 |
|  | 1.2180 | 1.1350 |
|  | 0.9700 | 1.0470 |
|  | 0.8680 | 0.4390 |
|  | 0.9960 | 0.8980 |
|  | 1.2390 | 0.9800 |
|  | 1.5690 | 1.3710 |
|  | 1.0250 | 0.6650 |
|  | 1.3720 | 1.1650 |
|  | 0.9040 | 0.7620 |
|  | 1.3290 | 0.9270 |
|  | 1.0280 | 1.8000 |
|  | 0.6480 | 0.6810 |
|  | 1.2650 | 0.6020 |
|  | 0.8510 | 0.8760 |
|  | 0.8880 | 0.7000 |
|  | 1.0210 | 0.7270 |
|  | 0.9360 | 0.5710 |
|  | 1.2650 | 0.4580 |
|  | 0.7270 | 0.7840 |
|  | 1.2790 | 0.8510 |
|  | 1.3540 | 0.7660 |
|  | 1.5990 | 0.8760 |
|  | 1.2990 | 0.8510 |
|  | 1.1570 | 1.3290 |
|  | 0.8380 | 1.6340 |
|  | 1.0350 | 0.5650 |
|  | 1.8050 | 1.4160 |
|  | 1.1570 | 0.9400 |
|  | 1.1570 | 0.6860 |
|  | 0.9700 | 1.7230 |
|  | 0.9510 | 0.9160 |
|  | 1.2560 | 0.6650 |
|  | 1.4460 | 0.9700 |
|  | 1.5340 | 1.3290 |
|  | 1.6170 | 0.9400 |
|  | 0.9920 | 1.1190 |
|  | 0.9510 | 0.7840 |
|  | 1.1350 | 1.3320 |
|  | 1.4460 | 1.6860 |
|  | 1.9120 | 0.9510 |
|  | 1.6950 | 1.4080 |
|  | 0.6020 | 0.8510 |
|  | 1.4490 | 0.6820 |
|  | 1.3640 | 0.9400 |
|  | 1.6170 | 1.1060 |
|  | 1.1350 | 1.2330 |
|  | 1.2560 | 0.7490 |
|  | 1.3372 | 1.5520 |
|  | 1.6720 | 0.8280 |
|  | 2.0580 | 0.8280 |
|  | 0.8380 | 1.3630 |
|  | 1.7320 | 1.1590 |
|  | 0.9510 | 1.0210 |
|  | 1.7230 | 0.6840 |
|  | 1.1910 | 0.8420 |
|  | 1.3210 | 1.5550 |
|  | 1.3610 | 0.8870 |
|  | 1.1190 | 1.0050 |
|  | 1.4160 | 0.9260 |
|  | 1.2990 | 1.1710 |
|  | 0.9200 | 0.8850 |
|  | 1.5640 | 1.0760 |
|  | 1.5690 | 1.1410 |
|  | 1.5220 | 1.2650 |
|  | 1.5990 | 0.8420 |
|  | 1.2650 | 1.3850 |
|  | 1.4460 | 1.3290 |
|  | 1.2650 | 1.3290 |
|  | 1.4260 | 1.6890 |
|  | 1.3020 | 1.2270 |
|  | 1.6340 | 1.3290 |
|  | 1.3450 | 0.9200 |
|  | 1.9420 | 0.9920 |
|  | 1.2560 | 1.2090 |
|  | 0.8030 | 0.9510 |
|  | 1.5990 | 1.3450 |
|  | 1.5220 | 1.3850 |
|  | 0.9200 | 1.1090 |
|  | 1.5640 | 0.9040 |
|  | 1.2990 | 1.0630 |
|  | 1.6260 | 1.2550 |
|  | 1.5690 | 1.8390 |
|  | 1.7310 | 2.1180 |
|  | 1.4810 | 1.1750 |
|  | 1.6230 | 0.8720 |
|  | 1.0640 | 1.0980 |
|  | 1.0030 | 1.7070 |
|  | 1.6200 | 1.1410 |
|  | 1.1450 | 1.1570 |
|  | 1.3990 | 1.5220 |
|  | 1.2130 | 1.3560 |
|  | 0.3360 | 0.8200 |
|  | 1.2130 | 0.7970 |
|  | 1.8080 | 1.1710 |
|  | 0.9810 | 1.5710 |
|  | 0.6810 | 1.3220 |
|  | 1.1500 | 1.7870 |
|  | 1.2760 | 0.8170 |
|  | 1.1750 | 0.9910 |
|  | 1.2400 | 1.2950 |
|  | 1.3450 | 1.1650 |
|  | 1.0850 | 1.1890 |
|  | 1.2400 | 0.9550 |
|  | 1.2030 | 0.8610 |
|  | 2.0430 | 1.7810 |
|  | 1.4810 | 1.3430 |
|  | 1.6650 | 1.3700 |
|  | 0.9510 | 1.1570 |
|  | 1.9140 | 1.1570 |
|  | 1.5950 | 0.7940 |
|  | 1.9170 | 1.3080 |
|  | 1.1700 | 1.2790 |
|  | 1.0640 | 1.4030 |
|  | 1.4310 | 0.8870 |
|  | 1.2400 | 1.2910 |
|  | 0.9510 | 2.0110 |
|  | 1.6230 | 1.2180 |
|  | 1.0950 | 1.2040 |
|  | 1.7050 | 0.5610 |
|  | 1.2400 | 1.0120 |
|  | 0.9150 | 1.0310 |
|  | 1.4930 | 1.1910 |
|  | 1.8110 | 0.9670 |
|  | 0.6380 | 1.7350 |
|  | 1.6610 | 1.0570 |
|  | 1.2400 | 1.3610 |
|  | 1.2400 | 1.3780 |
|  | 1.5810 | 0.7970 |
|  | 1.2760 | 0.6090 |
|  | 1.3990 | 1.0370 |
|  | 1.1700 | 0.9550 |
|  | 1.1890 | 1.3460 |
|  | 0.7440 | 1.4560 |
|  | 1.5950 | 1.0210 |
|  | 1.8080 | 0.8510 |
|  | 1.7020 | 1.1910 |
|  | 1.1890 | 1.2650 |
|  | 1.4930 | 1.1090 |
|  | 2.0240 | 1.5690 |
|  | 0.8770 | 1.2180 |
|  | 0.8310 | 1.1940 |
|  | 1.1940 | 0.5110 |
|  | 1.2880 | 1.5300 |
|  | 1.6950 | 1.7350 |
|  | 1.6260 | 1.0210 |
|  | 1.1090 | 1.6450 |
|  | 1.1090 | 1.6560 |
|  | 1.3320 | 1.5710 |
|  | 1.2030 | 1.6020 |
|  | 0.7840 | 1.2950 |
|  | 0.7660 | 1.3780 |
|  | 1.2030 | 1.8730 |
|  | 1.5340 | 1.2270 |
|  | 1.1060 | 1.2910 |
|  | 1.3640 | 0.6280 |
|  | 1.1310 | 1.2090 |
|  | 1.2980 | 1.2270 |
|  | 1.6230 | 1.1820 |
|  | 1.5700 | 1.5050 |
|  | 1.5040 | 0.7330 |
|  | 1.2450 | 0.9750 |
|  | 1.3450 | 1.0630 |
|  | 1.0640 | 0.8310 |
|  | 1.5040 | 0.9670 |
|  | 1.5700 | 0.9310 |
|  | 1.6820 | 0.8200 |
|  | 1.2400 | 1.0650 |
|  | 1.1500 | 0.9260 |
|  | 1.8080 | 0.8950 |
|  | 0.9150 | 1.3630 |
|  | 1.5950 | 1.5220 |
|  | 1.2810 | 1.2790 |
|  | 1.5120 | 0.7520 |
|  | 1.8110 | 0.7110 |
|  | 1.4310 | 0.8870 |
|  | 1.6200 | 1.0210 |
|  | 1.1100 | 1.7180 |
|  | 1.5230 | 0.7490 |
|  | 1.1450 | 0.8310 |
|  | 0.9150 | 0.7940 |
|  | 1.1570 | 0.8800 |
|  | 0.8760 | 0.9160 |
|  | 1.1090 | 0.8660 |
|  | 1.1420 | 1.7350 |
|  | 1.2090 | 0.9670 |
|  | 0.8510 | 1.3770 |
|  | 1.6370 | 1.6840 |
|  | 0.9160 | 0.9630 |
|  | 1.5340 | 1.7000 |
|  | 0.7700 | 1.2270 |
|  | 1.2090 | 1.6650 |
|  | 0.6860 | 1.7110 |
|  | 1.1090 | 1.1500 |
|  | 1.1910 | 0.8310 |
|  | 0.8030 | 1.0090 |
|  | 0.7700 | 1.2850 |
|  | 0.9510 | 0.8100 |
|  | 1.2650 | 1.0640 |
|  | 1.6430 | 1.0640 |
|  | 0.9200 | 0.9810 |
|  | 1.2880 | 1.0950 |
|  | 0.9700 | 1.0640 |
|  | 0.9160 | 0.7310 |
|  | 1.3450 | 1.0640 |
|  | 1.6430 | 1.0650 |
|  | 1.1090 | 1.1650 |
|  | 1.2650 | 1.7310 |
|  | 1.3640 | 0.8570 |
|  | 1.0830 | 0.6200 |
|  | 0.8510 | 0.6380 |
|  | 1.3240 | 0.9570 |
|  | 0.9700 | 1.1890 |
|  | 1.0830 | 0.7130 |
|  | 1.2760 | 1.0640 |
|  | 1.2560 | 1.5810 |
|  | 0.8760 | 1.2030 |
|  | 0.9040 | 1.2030 |
|  | 0.6860 | 0.8100 |
|  | 1.0660 | 0.6200 |
|  | 1.0210 | 1.3620 |
|  | 1.4460 | 1.0640 |
|  | 0.8550 | 0.8310 |
|  | 1.2510 | 1.4780 |
|  | 0.9550 | 1.1500 |
|  | 0.7760 | 1.1310 |
|  | 1.1230 | 1.5040 |
|  | 0.9910 | 1.0470 |
|  | 1.5660 | 1.0470 |
|  | 1.0410 | 1.0030 |
|  | 1.1270 | 0.6810 |
|  | 0.9460 | 1.0640 |
|  | 1.1630 | 0.9810 |
|  | 1.1750 | 1.1890 |
|  | 1.1910 | 1.2130 |
|  | 1.5220 | 1.0470 |
|  | 1.1590 | 1.4190 |
|  | 1.4360 | 1.3830 |
|  | 0.8280 | 1.1890 |
|  | 0.7760 | 1.7310 |
|  | 0.8720 | 0.9630 |
|  | 1.0910 | 1.7510 |
|  | 0.6940 | 0.9810 |
|  | 0.6860 | 1.1750 |
|  | 1.2960 | 0.9920 |
|  | 1.4490 | 2.3410 |
|  | 1.2270 | 1.4030 |
|  | 1.0900 | 1.2180 |
|  | 1.0900 | 1.1850 |
|  | 1.2790 | 0.9700 |
|  | 1.4490 | 1.2270 |
|  | 1.0250 | 1.5080 |
|  | 1.1060 | 0.7270 |
|  | 2.2140 | 0.9700 |
|  | 1.1820 | 1.1850 |
|  | 0.9920 | 1.8800 |
|  | 0.9400 | 0.9700 |
|  | 1.4260 | 1.1570 |
|  | 1.1190 | 0.8880 |
|  | 1.1940 | 0.7840 |
|  | 1.0350 | 0.9920 |
|  | 0.9360 | 1.4460 |
|  | 1.0250 | 1.8050 |
|  | 0.8760 | 0.8880 |
|  | 1.1570 | 2.1270 |
|  | 0.9400 | 1.0250 |
|  | 1.0660 | 1.4460 |
|  | 1.1910 | 1.4860 |
|  | 1.1450 | 1.1090 |
|  | 1.1090 | 1.2650 |
|  | 0.9360 | 1.6050 |
|  | 0.9360 | 1.0660 |
|  | 1.1090 | 1.6390 |
|  | 0.6020 | 1.1330 |
|  | 1.1910 | 1.2180 |
|  | 1.0250 | 1.1080 |
|  | 1.1090 | 1.0300 |
|  | 0.9400 | 1.1890 |
|  | 0.8550 | 1.2330 |
|  | 1.2880 | 1.2500 |
|  | 1.0250 | 1.2550 |
|  | 0.8550 | 1.0630 |
|  | 1.3020 | 0.8420 |
|  | 0.8880 | 0.7110 |
|  | 1.3750 | 1.1230 |
|  | 1.1570 | 1.1910 |
|  | 0.7770 | 1.1890 |
|  | 1.3750 | 1.6810 |
|  | 1.1570 | 1.1000 |
|  | 0.5860 | 2.1750 |
|  | 1.4970 | 1.0050 |
|  | 1.4290 | 0.5820 |
|  | 1.2550 | 0.9670 |
|  | 1.2420 | 0.8200 |
|  | 0.9910 | 1.7030 |
|  | 0.7330 | 1.1330 |
|  | 1.5540 | 0.9630 |
|  | 1.3610 | 1.4310 |
|  | 1.0980 | 1.4270 |
|  | 1.0050 | 1.2030 |
|  | 1.0760 | 1.2030 |
|  | 1.0760 | 0.8100 |
|  | 1.0370 | 0.9090 |
|  | 0.6420 | 1.5950 |
|  | 1.5070 | 1.2810 |
|  | 1.7230 | 1.0090 |
|  | 1.8460 | 0.9150 |
|  | 1.0210 | 0.8510 |
|  | 1.1750 | 2.0320 |
|  | 0.8870 | 0.9810 |
|  | 1.9290 | 1.1890 |
|  | 1.2560 | 1.0600 |
|  | 1.3860 | 1.5230 |
|  | 1.3850 | 1.0090 |
|  | 0.9200 | 1.6270 |
|  | 0.7840 | 1.5990 |
|  | 1.3850 | 1.1310 |
|  | 1.1350 | 1.1310 |
|  | 1.5990 | 1.3540 |
|  | 1.0280 | 1.5340 |
|  | 0.5960 | 1.8820 |
|  | 1.1090 | 1.4310 |
|  | 1.3210 | 1.0640 |
|  | 1.6760 | 1.7150 |
|  | 0.9510 | 1.0640 |
|  | 1.5340 | 1.4310 |
|  | 1.7120 | 1.1310 |
|  | 1.5990 | 1.2650 |
|  | 1.1850 | 1.2130 |
|  | 1.4160 | 1.0210 |
|  | 1.4080 | 1.8740 |
|  | 1.1570 | 1.5340 |
|  | 1.1570 | 1.2030 |
|  | 1.1060 | 1.6650 |
|  | 1.4560 | 0.4860 |
|  | 1.6190 | 1.6200 |
|  | 1.0760 | 1.6230 |
|  | 0.9700 | 1.0690 |
|  | 0.7610 | 1.0850 |
|  | 1.2030 | 1.2810 |
|  | 1.2990 | 1.2810 |
|  | 1.4260 | 1.2940 |
|  | 0.7610 | 1.0530 |
|  | 1.0850 | 0.7670 |
|  | 1.7390 | 0.9150 |
|  | 1.2660 | 1.0640 |
|  | 1.4360 | 1.4930 |
|  | 1.4360 | 1.7050 |
|  | 0.9160 | 0.6810 |
|  | 1.2030 | 0.7440 |
|  | 1.2990 | 1.2760 |
|  | 1.6520 | 1.8210 |
|  | 1.5340 | 1.5040 |
|  | 1.1090 | 1.9140 |
|  | 1.1570 | 1.7050 |
|  | 0.9360 | 1.2450 |
|  | 1.1090 | 1.7150 |
|  | 1.3240 | 1.7540 |
|  | 0.9960 | 2.5030 |
|  | 0.9040 | 1.6270 |
|  | 1.1090 | 1.8570 |
|  | 1.9860 | 2.0180 |
|  | 1.1540 | 1.9610 |
|  | 0.9200 | 1.5490 |
|  | 1.3290 | 1.7830 |
|  | 1.7700 | 1.5230 |
|  | 0.6140 | 1.2130 |
|  | 0.8510 | 0.9510 |
|  | 0.7610 | 1.0690 |
|  | 1.2180 | 1.3870 |
|  | 1.3720 | 1.3160 |
|  | 1.1570 | 1.1890 |
|  | 0.4250 | 1.8300 |
|  | 1.4490 | 1.3850 |
|  | 1.2650 | 1.2560 |
|  | 0.9700 | 0.8880 |
|  | 1.0280 | 0.9920 |
|  | 0.9920 | 0.7840 |
|  | 0.8380 | 1.1910 |
|  | 1.2650 | 1.2650 |
|  | 1.2650 | 0.9630 |
|  | 1.5690 | 1.1420 |
|  | 1.2650 | 1.1940 |
|  | 1.3720 | 0.9700 |
|  | 1.0830 | 1.1940 |
|  | 1.1450 | 1.1420 |
|  | 1.0390 | 1.1450 |
|  | 1.5150 | 0.7700 |
|  | 1.5410 | 0.8880 |
|  | 1.1060 | 1.1510 |
|  | 0.8380 | 1.6260 |
|  | 1.2650 | 1.2270 |
|  | 1.2960 | 1.3850 |
|  | 0.8680 | 1.6260 |
|  | 1.2880 | 1.6430 |
|  | 1.7700 | 1.3720 |
|  | 1.1570 | 1.4540 |
|  | 0.7020 | 1.1450 |
|  | 1.2030 | 0.7610 |
|  | 1.6780 | 1.6860 |
|  | 1.0760 | 2.0490 |
|  | 1.3750 | 0.9510 |
|  | 0.8070 | 0.7840 |
|  | 1.1060 | 1.4640 |
|  | 0.7660 | 1.2990 |
|  | 1.4540 | 2.1130 |
|  | 1.7230 | 1.7460 |
|  | 1.7520 | 1.5830 |
|  | 1.0250 | 0.7840 |
|  | 1.1450 | 1.1940 |
|  | 1.2270 | 1.1910 |
|  | 0.6480 | 1.4860 |
|  | 1.1450 | 1.9200 |
|  | 1.4880 | 1.2650 |
|  | 1.4490 | 1.2560 |
|  | 1.1450 | 1.7540 |
|  | 1.7460 | 0.7610 |
|  | 1.3290 | 0.8380 |
|  | 1.3950 | 1.0250 |
|  | 1.0830 | 1.5690 |
|  | 1.4560 | 1.2390 |
|  | 1.7350 | 1.2180 |
|  | 1.1940 | 0.9160 |
|  | 1.1570 | 1.6090 |
|  | 1.6890 | 1.5350 |
|  | 1.4560 | 1.0210 |
|  | 1.5340 | 1.8060 |
|  | 1.5690 | 1.0120 |
|  | 1.0760 | 0.9510 |
|  | 0.9960 | 1.1850 |
|  | 0.8680 | 0.9920 |
|  | 1.1060 | 1.3850 |
|  | 1.4440 | 1.6260 |
|  | 1.2880 | 1.2760 |
|  | 1.5340 | 1.1420 |
|  | 1.4460 | 1.4490 |
|  | 1.5640 | 0.7320 |
|  | 1.3240 | 0.8550 |
|  | 1.3200 | 1.2180 |
|  | 1.5640 | 1.2650 |
|  | 1.9840 | 0.7700 |
|  | 1.4560 | 1.1940 |
|  | 1.1820 | 0.6810 |
|  | 1.4880 | 0.7840 |
|  | 0.6810 | 1.1570 |
|  | 1.2390 | 0.7840 |
|  | 1.1820 | 0.9400 |
|  | 1.1570 | 1.0210 |
|  | 1.4310 | 1.0210 |
|  | 0.6470 | 1.1090 |
|  | 1.1890 | 1.1450 |
|  | 1.7050 | 1.0250 |
|  | 1.5040 | 1.0030 |
|  | 0.8510 | 1.5700 |
|  | 1.4270 | 1.5700 |
|  | 1.8360 | 2.2330 |
|  | 1.2130 | 1.0690 |
|  | 1.1100 | 1.0640 |
|  | 1.9550 | 1.4310 |
|  | 1.6270 | 2.0460 |
|  | 1.5530 | 1.0470 |
|  | 1.2180 | 1.0030 |
|  | 1.4860 | 1.2130 |
|  | 1.7540 | 1.2880 |
|  | 1.6850 | 1.7020 |
|  | 1.3850 | 1.4490 |
|  | 1.3000 | 0.9510 |
|  | 1.4490 | 0.9960 |
|  | 0.9920 | 1.1420 |
|  | 1.3290 | 0.9510 |
|  | 0.8760 | 1.3290 |
|  | 2.2270 | 1.8800 |
|  | 1.0900 | 1.0980 |
|  | 1.3290 | 1.2930 |
|  | 1.0390 | 1.8160 |
|  | 1.6520 | 1.2500 |
|  | 1.8050 | 1.5670 |
|  | 1.4260 | 1.5710 |
|  | 2.0080 | 0.9130 |
|  | 1.2180 | 1.2550 |
|  | 0.5960 | 1.0050 |
|  | 1.1910 | 1.3780 |
|  | 1.3450 | 0.8850 |
|  | 0.8550 | 1.0760 |
|  | 0.6020 | 1.2060 |
|  | 1.7040 | 1.0390 |
|  | 0.9360 | 1.7070 |
|  | 1.0390 | 0.9080 |
|  | 1.6740 | 1.3750 |
|  | 1.1710 | 1.2880 |
|  | 1.2510 | 1.2650 |
|  | 1.5520 | 0.9920 |
|  | 1.1910 | 1.5830 |
|  | 1.3000 | 1.6190 |
|  | 0.7700 | 0.7320 |
|  | 0.9670 | 1.4160 |
|  | 1.0120 | 2.1520 |
|  | 1.0630 | 1.6780 |
|  | 1.1080 | 1.2650 |
|  | 1.0760 | 0.7700 |
|  | 1.1910 | 1.6260 |
|  | 1.4440 | 0.9920 |
|  | 1.5890 | 0.6190 |
|  | 1.1230 | 1.5990 |
|  | 1.2040 | 0.5180 |
|  | 1.7350 | 1.1060 |
|  | 1.0650 | 1.2790 |
|  | 0.8200 | 1.4260 |
|  | 1.2720 | 0.7700 |
|  | 0.9670 | 0.6860 |
|  | 0.8720 | 1.7310 |
|  | 0.7330 | 1.5700 |
|  | 1.2550 | 1.2130 |
|  | 1.0120 | 1.2130 |
|  | 1.9030 | 1.5810 |
|  | 1.7050 | 1.6650 |
|  | 1.3000 | 2.0240 |
|  | 1.3830 | 1.4930 |
|  | 1.7310 | 1.0640 |
|  | 1.5040 | 1.4810 |
|  | 1.7310 | 1.2810 |
|  | 1.3830 | 1.2130 |
|  | 0.9090 | 1.8360 |
|  | 1.5230 | 0.7740 |
|  | 1.1890 | 1.2400 |
|  | 1.9030 | 1.2270 |
|  | 0.9150 | 1.6760 |
|  | 1.8300 | 1.2650 |
|  | 1.8110 | 1.4260 |
|  | 1.0640 | 0.9200 |
|  | 1.3330 | 1.7460 |
|  | 1.0470 | 1.4690 |
|  | 1.2980 | 0.7660 |
|  | 0.7670 | 0.9510 |
|  | 1.6610 | 1.1190 |
|  | 1.0090 | 1.4460 |
|  | 1.0850 | 1.7040 |
|  | 1.0640 | 0.7700 |
|  | 1.0640 | 1.1090 |
|  | 1.4810 | 0.8550 |
|  | 2.1400 | 0.7700 |
|  | 0.9360 | 0.9700 |
|  | 1.4890 | 1.0900 |
|  | 1.0690 | 1.1570 |
|  | 1.1060 | 0.7700 |
|  | 1.1090 | 1.2800 |
|  | 0.9160 | 1.0390 |
|  | 2.3840 | 1.1940 |
|  | 0.6650 | 1.6370 |
|  | 1.6890 | 0.8070 |
|  | 1.2790 | 0.7670 |
|  | 1.2650 | 1.8170 |
|  | 1.0250 | 1.5700 |
|  | 0.9400 | 1.5230 |
|  | 1.4080 | 1.6820 |
|  | 0.9920 | 0.7670 |
|  | 1.0900 | 1.3450 |
|  | 1.2090 | 1.1500 |
|  | 1.0202 | 1.6230 |
|  | 0.8510 | 1.3330 |
|  | 1.3850 | 1.4470 |
|  | 1.3950 | 1.2400 |
|  | 0.9040 | 1.8890 |
|  | 1.0900 | 1.2810 |
|  | 1.0900 | 1.8570 |
|  | 0.6860 | 1.2390 |
|  | 1.1570 | 0.9360 |
|  | 1.1460 | 1.2980 |
|  | 0.6860 | 1.3210 |
|  | 0.8420 | 1.6510 |
|  | 1.3720 | 1.1090 |
|  | 0.7020 | 1.0690 |
|  | 0.6860 | 0.9570 |
|  | 1.3640 | 1.6510 |
|  | 2.1790 | 1.2130 |
|  | 0.7720 | 1.2290 |
|  | 0.6020 | 1.2290 |
|  | 1.6520 | 0.9830 |
|  | 1.3720 | 1.6000 |
|  | 1.1090 | 1.2290 |
|  | 1.3450 | 0.8600 |
|  | 1.3850 | 1.1060 |
|  | 1.0390 | 1.4800 |
|  | 1.6190 | 0.7700 |
|  | 1.6190 | 1.2530 |
|  | 0.9400 | 1.5970 |
|  | 0.9040 | 0.8240 |
|  | 1.0390 | 1.1330 |
|  | 1.2960 | 0.8600 |
|  | 2.0490 | 1.8470 |
|  | 1.2130 | 1.4800 |
|  | 1.7180 | 1.1330 |
|  | 1.1890 | 0.7870 |
|  | 1.1500 | 1.0990 |
|  | 1.2130 | 1.7070 |
|  | 1.5340 | 1.1060 |
|  | 2.2130 | 1.1130 |
|  | 1.0950 | 1.1130 |
|  | 1.3330 | 1.6020 |
|  | 1.8570 | 1.4010 |
|  | 1.9170 | 1.0470 |
|  | 1.1450 | 0.9200 |
|  | 1.0090 | 1.2430 |
|  | 1.2810 | 1.7270 |
|  | 1.4470 | 1.1610 |
|  | 1.7180 | 0.8360 |
|  | 1.2850 | 0.9470 |
|  | 1.3830 | 0.9570 |
|  | 1.5080 | 1.5020 |
|  | 0.9160 | 1.0490 |
|  | 0.7700 | 1.2210 |
|  | 0.7610 | 1.3370 |
|  | 1.6050 | 1.1120 |
|  | 0.6650 | 1.3760 |
|  | 0.8000 | 1.0930 |
|  | 0.8550 | 0.7420 |
|  | 0.7840 | 0.7860 |
|  | 1.0210 | 0.6720 |
|  | 1.6370 | 1.2800 |
|  | 1.7950 | 1.3580 |
|  | 1.5220 | 1.2800 |
|  | 1.1820 | 1.1120 |
|  | 1.6430 | 0.7860 |
|  | 0.7840 | 0.9730 |
|  | 1.4690 | 1.2900 |
|  | 1.5530 | 1.2900 |
|  | 1.0250 | 1.1820 |
|  | 1.0456 | 1.3390 |
|  | 1.1190 | 0.6720 |
|  | 1.2390 | 1.0000 |
|  | 1.2650 | 1.3090 |
|  | 1.8270 | 1.3760 |
|  | 0.9400 | 1.4500 |
|  | 1.1190 | 1.1690 |
|  | 1.6370 | 1.0700 |
|  | 1.7350 | 1.4650 |
|  | 1.0900 | 0.9200 |
|  | 0.7320 | 1.0390 |
|  | 1.0260 | 0.9570 |
|  | 0.7520 | 1.7180 |
|  | 0.9020 | 0.6670 |
|  | 0.9020 | 1.1010 |
|  | 0.9020 | 0.6290 |
|  | 1.2810 | 2.1100 |
|  | 0.6810 | 1.5730 |
|  | 0.6810 | 1.1260 |
|  | 0.8970 | 0.8100 |
|  | 0.9810 | 1.5270 |
|  | 1.4470 | 1.5920 |
|  | 1.4810 | 1.9020 |
|  | 1.1700 | 1.1340 |
|  | 1.6090 | 1.1340 |
|  | 1.3620 | 0.7790 |
|  | 0.7670 | 0.8360 |
|  | 1.2980 | 0.7030 |
|  | 1.5340 | 0.7250 |
|  | 1.5230 | 1.2610 |
|  | 1.2270 | 0.8790 |
|  | 1.3290 | 0.8100 |
|  | 1.0900 | 1.2430 |
|  | 1.2030 | 0.9470 |
|  | 1.6260 | 0.9850 |
|  | 1.2270 | 0.9730 |
|  | 1.3290 | 1.0580 |
|  | 1.1090 | 0.7460 |
|  | 1.3720 | 0.7250 |
|  | 1.1420 | 1.4920 |
|  | 0.8510 | 0.7860 |
|  | 1.7020 | 0.6140 |
|  | 0.8880 | 1.2430 |
|  | 1.7020 | 1.3390 |
|  | 0.9200 | 1.4240 |
|  | 1.2270 | 1.1820 |
|  | 1.3290 | 1.0250 |
|  | 0.9630 | 0.7420 |
|  | 1.6260 | 0.9440 |
|  | 1.1820 | 0.9440 |
|  | 1.3850 | 1.3460 |
|  | 0.9630 | 1.2680 |
|  | 1.1080 | 1.2800 |
|  | 1.1650 | 1.1180 |
|  | 1.0390 | 0.9440 |
|  | 1.0890 | 1.2580 |
|  | 0.5860 | 0.9850 |
|  | 0.4810 | 0.8600 |
|  | 2.0240 | 1.1010 |
|  | 1.1890 | 1.1260 |
|  | 1.3090 | 1.6890 |
|  | 0.8870 | 0.9850 |
|  | 1.3080 | 1.4500 |
|  | 1.1890 | 1.0340 |
|  | 0.9080 | 0.7120 |
|  | 1.6810 | 1.2030 |
|  | 0.6460 | 1.7460 |
|  | 0.8420 | 1.1900 |
|  | 0.8530 | 0.8100 |
|  | 1.0910 | 1.0220 |
|  | 1.0910 | 0.8360 |
|  | 1.0910 | 0.5750 |
|  | 1.7710 | 1.6590 |
|  | 1.5070 | 1.2680 |
|  | 1.2990 | 1.4440 |
|  | 1.7120 | 0.7460 |
|  | 0.7840 | 0.9600 |
|  | 1.1420 | 1.3530 |
|  | 0.9920 | 0.8510 |
|  | 1.2560 | 1.2210 |
|  | 0.3970 | 0.8100 |
|  | 0.9820 | 1.2680 |
|  | 1.1160 | 1.4460 |
|  | 1.3080 | 0.7460 |
|  | 1.0830 | 1.3530 |
|  | 0.4960 | 0.9170 |
|  | 0.4810 | 0.8470 |
|  | 0.9160 | 0.7790 |
|  | 0.9630 | 0.9470 |
|  | 0.9260 | 0.8360 |
|  | 1.3680 | 0.8100 |
|  | 1.8330 | 1.2000 |
|  | 1.3090 | 0.6920 |
|  | 1.5890 | 0.8830 |
|  | 0.7520 | 1.0710 |
|  | 1.0120 | 0.6920 |
|  | 1.0050 | 0.5970 |
|  | 0.9160 | 0.6570 |
|  | 1.5220 | 0.9460 |
|  | 1.4820 | 0.8830 |
|  | 1.5110 | 1.2460 |
|  | 0.7760 | 1.0710 |
|  | 1.8830 | 0.6420 |
|  | 2.1970 | 0.7340 |
|  | 1.1410 | 0.9950 |
|  | 1.0410 | 1.0470 |
|  | 0.9480 | 0.9580 |
|  | 1.0630 | 0.6290 |
|  | 0.9750 | 1.1250 |
|  | 1.4160 | 0.9520 |
|  | 1.4470 | 1.0700 |
|  | 2.3390 | 1.2720 |
|  | 1.0120 | 0.5700 |
|  | 0.8200 | 1.0140 |
|  | 0.8200 | 0.6420 |
|  | 1.0210 | 1.1700 |
|  | 1.1590 | 0.6320 |
|  | 0.6460 | 0.8810 |
|  | 0.8870 | 0.9460 |
|  | 1.1270 | 1.1570 |
|  | 0.9260 | 0.9950 |
|  | 1.3090 | 1.0380 |
|  | 0.5780 | 0.8200 |
|  | 1.5430 | 0.9620 |
|  | 1.2660 | 1.0860 |
|  | 1.2510 | 0.7550 |
|  | 1.4690 | 0.4790 |
|  | 1.5890 | 0.6480 |
|  | 1.4550 | 1.5840 |
|  | 1.6460 | 1.2020 |
|  | 1.1570 | 0.9350 |
|  | 0.8280 | 0.8810 |
|  | 0.6160 | 0.9850 |
|  | 0.7490 | 1.1690 |
|  | 0.8950 | 0.6490 |
|  | 1.2270 | 0.8020 |
|  | 0.8420 | 1.0700 |
|  | 1.0980 | 1.1260 |
|  | 0.9630 | 1.6940 |
|  | 1.4310 | 1.3760 |
|  | 1.3630 | 0.7080 |
|  | 0.8850 | 0.3930 |
|  | 1.3000 | 0.7460 |
|  | 1.1590 | 1.0930 |
|  | 0.5610 | 0.9470 |
|  | 0.6940 | 0.8360 |
|  | 0.7610 | 0.7860 |
|  | 0.7610 | 0.5670 |
|  | 1.7750 | 0.8020 |
|  | 1.0890 | 1.3910 |
|  | 2.0520 | 1.0580 |
|  | 1.5790 | 0.7550 |
|  | 1.0890 | 0.9170 |
|  | 1.4870 | 1.0700 |
|  | 1.0760 | 1.4180 |
|  | 1.2660 | 1.0950 |
|  | 1.1590 | 1.1450 |
|  | 1.1590 | 0.9470 |
|  | 1.3560 | 1.3180 |
|  | 1.4570 | 1.4690 |
|  | 1.1160 | 0.6140 |
|  | 1.2660 | 1.0930 |
|  | 0.7010 | 0.5730 |
|  | 0.6420 | 0.7460 |
|  | 1.2840 | 1.7460 |
|  | 1.2790 | 1.0250 |
|  | 0.9910 | 1.0950 |
|  | 1.5950 | 1.7740 |
|  | 1.2660 | 0.9440 |
|  | 1.9030 | 1.4290 |
|  | 1.6810 | 1.2610 |
|  | 0.7360 | 0.7080 |
|  | 0.8870 | 1.1800 |
|  | 1.7070 | 1.3020 |
|  | 1.0650 | 0.9950 |
|  | 1.4930 | 1.1260 |
|  | 1.0910 | 1.1820 |
|  | 1.3560 | 0.4720 |
|  | 1.0230 | 1.1980 |
|  | 1.0230 | 1.4240 |
|  | 1.2270 | 0.7030 |
|  | 0.6840 | 0.7120 |
|  | 1.0390 | 1.5270 |
|  | 0.7840 | 1.4840 |
|  | 1.2090 | 0.9470 |
|  | 1.3450 | 1.4460 |
|  | 0.7610 | 0.9600 |
|  | 1.0280 | 0.7750 |
|  | 0.4570 | 1.2280 |
|  | 0.6560 | 1.9660 |
|  | 1.0400 | 1.1820 |
|  | 0.8520 | 1.5020 |
|  | 0.7100 | 0.7080 |
|  | 0.6580 | 1.5190 |
|  | 0.4150 | 1.2210 |
|  | 0.6560 | 1.3760 |
|  | 1.8300 | 0.9440 |
|  | 0.8310 | 2.0460 |
|  | 0.8170 | 1.2800 |
|  | 1.1580 | 1.0250 |
|  | 1.2240 | 1.0700 |
|  | 1.2350 | 1.0700 |
|  | 0.4800 | 1.0700 |
|  | 0.5020 | 0.7080 |
|  | 1.4380 | 1.0580 |
|  | 1.0010 | 1.2900 |
|  | 1.1990 | 1.2970 |
|  | 0.9400 | 0.7080 |
|  | 0.7330 | 1.1900 |
|  | 0.9630 | 1.1180 |
|  | 0.5790 | 0.7860 |
|  | 0.9510 | 1.3390 |
|  | 0.6840 | 1.4780 |
|  | 1.6340 | 0.7030 |
|  | 1.5000 | 0.9200 |
|  | 1.1000 | 0.9440 |
|  | 1.1910 | 1.0220 |
|  | 1.0980 | 1.0250 |
|  | 2.0310 | 1.2610 |
|  | 0.7970 | 1.2230 |
|  | 0.7940 | 1.9040 |
|  | 0.5320 | 1.2430 |
|  | 0.7310 | 1.3390 |
|  | 1.1230 | 1.4840 |
|  | 1.3880 | 1.1610 |
|  | 1.3610 | 0.8790 |
|  | 1.3950 | 0.8020 |
|  | 0.5890 | 0.9600 |
|  | 1.3480 | 1.0340 |
|  | 1.3170 | 1.2000 |
|  | 1.1580 | 1.3090 |
|  | 1.1730 | 1.4350 |
|  | 1.4790 | 1.1690 |
|  | 1.2570 | 1.4460 |
|  | 1.1640 | 1.0700 |
|  | 0.6560 | 1.4160 |
|  | 0.8720 | 0.8360 |
|  | 0.8760 | 0.5280 |
|  | 0.9700 | 0.9600 |
|  | 1.3090 | 1.3760 |
|  | 0.7360 | 0.7420 |
|  | 0.8870 | 1.5020 |
|  | 0.9530 | 1.1610 |
|  | 1.4610 | 1.5610 |
|  | 0.9530 | 1.2230 |
|  | 1.0630 | 0.6770 |
|  | 1.1000 | 1.2230 |
|  | 0.7240 | 1.1900 |
|  | 0.5780 | 0.9950 |
|  | 0.7760 | 1.0930 |
|  | 1.3780 | 1.3440 |
|  | 1.2330 | 1.6190 |
|  | 0.8220 | 1.0700 |
|  | 1.1330 | 0.9010 |
|  | 1.7060 | 1.5220 |
|  | 0.9080 | 1.0090 |
|  | 0.8220 | 0.6230 |
|  | 1.0370 | 0.4580 |
|  | 1.5830 | 1.1950 |
|  | 0.5490 | 1.4140 |
|  | 0.9460 | 1.1820 |
|  | 0.9670 | 0.3830 |
|  | 1.0760 | 0.8560 |
|  | 1.1550 | 0.6320 |
|  | 1.0120 | 1.5150 |
|  | 1.2270 | 1.3840 |
|  | 1.0210 | 0.6920 |
|  | 1.0370 | 1.1550 |
|  | 1.7060 | 0.5410 |
|  | 1.0050 | 1.1950 |
|  | 0.8310 | 1.3450 |
|  | 1.3610 | 0.3830 |
|  | 0.6420 | 0.8810 |
|  | 0.7010 | 0.6320 |
|  | 0.4300 | 0.7550 |
|  | 1.0980 | 0.7650 |
|  | 1.0590 | 1.1970 |
|  | 0.8610 | 0.8200 |
|  | 0.5320 | 1.1480 |
|  | 0.6810 | 1.0380 |
|  | 0.7760 | 1.4950 |
|  | 0.6810 | 0.6780 |
|  | 0.5860 | 1.2830 |
|  | 1.6810 | 0.7580 |
|  | 0.6810 | 0.6690 |
|  | 1.1630 | 1.1750 |
|  | 0.8870 | 0.8180 |
|  | 0.6810 | 1.1820 |
|  | 0.9260 | 0.9770 |
|  | 0.7240 | 0.8760 |
|  | 1.4470 | 0.6420 |
|  | 0.8220 | 0.8280 |
|  | 0.9480 | 1.2100 |
|  | 0.7240 | 1.0700 |
|  | 0.7660 | 0.9010 |
|  | 0.9510 | 1.5150 |
|  | 0.7840 | 1.5130 |
|  | 1.2560 | 0.8560 |
|  | 0.8680 | 1.2430 |
|  | 0.9920 | 0.8460 |
|  | 1.0520 | 1.0990 |
|  | 0.7270 | 0.7680 |
|  | 0.7320 | 1.0590 |
|  | 0.9920 | 0.9330 |
|  | 1.2990 | 1.7590 |
|  | 1.0660 | 1.0260 |
|  | 1.0760 | 1.0860 |
|  | 1.1820 | 0.9060 |
|  | 1.0280 | 0.9880 |
|  | 1.1570 | 0.8850 |
|  | 0.6860 | 0.2820 |
|  | 0.9510 | 0.7860 |
|  | 0.4510 | 0.8110 |
|  | 1.2560 | 2.7090 |
|  | 1.1420 | 1.4970 |
|  | 0.7660 | 1.4970 |
|  | 0.4960 | 0.9270 |
|  | 0.7840 | 0.4920 |
|  | 0.8510 | 0.6590 |
|  | 1.4560 | 0.7680 |
|  | 1.0210 | 1.4070 |
|  | 0.9160 | 1.4310 |
|  | 0.9920 | 1.2320 |
|  | 1.0760 | 0.5730 |
|  | 1.1450 | 0.6590 |
|  | 0.8070 | 1.3760 |
|  | 1.2500 | 1.0810 |
|  | 0.8200 | 1.4750 |
|  | 1.0650 | 0.8900 |
|  | 1.6480 | 1.0260 |
|  | 1.5110 | 1.1510 |
|  | 1.4030 | 1.3190 |
|  | 1.2180 | 1.7720 |
|  | 1.1630 | 0.6590 |
|  | 0.7940 | 1.4970 |
|  | 1.5570 | 1.2780 |
|  | 0.8870 | 0.7930 |
|  | 2.4000 | 1.2160 |
|  | 0.8850 | 1.0590 |
|  | 1.0910 | 0.9680 |
|  | 1.4160 | 1.5540 |
|  | 0.7300 | 1.3120 |
|  | 1.2180 | 1.1880 |
|  | 0.8220 | 1.7390 |
|  | 1.0980 | 1.3970 |
|  | 0.9160 | 1.1840 |
|  | 0.8660 | 1.3370 |
|  | 0.5320 | 1.6360 |
|  | 0.6280 | 1.1210 |
|  | 0.7970 | 0.9680 |
|  | 1.2840 | 0.9830 |
|  | 1.4660 | 0.8460 |
|  | 1.3000 | 0.7680 |
|  | 0.9820 | 1.2320 |
|  | 1.2120 | 1.3650 |
|  | 1.1910 | 1.2510 |
|  | 1.2510 | 0.7680 |
|  | 0.9130 | 2.3630 |
|  | 0.8100 | 1.5010 |
|  | 1.2180 | 1.4070 |
|  | 0.8610 | 1.2000 |
|  | 1.5500 | 0.6290 |
|  | 0.8610 | 0.8110 |
|  | 1.2270 | 0.7090 |
|  | 1.0210 | 1.6710 |
|  | 1.3630 | 1.3690 |
|  | 0.8950 | 1.5260 |
|  | 1.4990 | 0.9270 |
|  | 1.2250 | 1.0030 |
|  | 1.3770 | 1.0990 |
|  | 1.4360 | 0.7090 |
|  | 1.0230 | 1.1840 |
|  | 1.3630 | 1.7170 |
|  | 0.9750 | 2.8300 |
|  | 1.1230 | 0.9880 |
|  | 1.1890 | 1.1151 |
|  | 1.3080 | 1.6000 |
|  | 0.8200 | 1.2000 |
|  | 0.8660 | 1.6000 |
|  | 1.0590 | 0.5900 |
|  | 0.7520 | 1.3660 |
|  | 1.1890 | 1.4510 |
|  | 1.4360 | 1.0260 |
|  | 1.2790 | 1.1180 |
|  | 0.8870 | 1.2580 |
|  | 1.2660 | 1.1010 |
|  | 1.4030 | 1.4160 |
|  | 1.3430 | 1.1260 |
|  | 1.0120 | 0.9440 |
|  | 1.2270 | 0.5560 |
|  | 1.2040 | 1.6210 |
|  | 1.3090 | 0.8790 |
|  | 1.6060 | 1.0220 |
|  | 1.2120 | 0.7860 |
|  | 0.8280 | 1.1040 |
|  | 1.7700 | 1.8110 |
|  | 1.6260 | 1.0700 |
|  | 1.5050 | 1.5730 |
|  | 1.4490 | 0.8650 |
|  | 1.2990 | 1.1040 |
|  | 2.2270 | 0.9440 |
|  | 1.1060 | 1.2030 |
|  | 1.4460 | 1.0930 |
|  | 1.0280 | 1.1800 |
|  | 0.9040 | 1.1260 |
|  | 1.0350 | 1.0220 |
|  | 0.9400 | 1.2000 |
|  | 0.8550 | 1.3090 |
|  | 0.8380 | 1.3730 |
|  | 0.8680 | 1.3910 |
|  | 1.3450 | 0.8650 |
|  | 1.7780 | 0.7420 |
|  | 1.2850 | 1.2280 |
|  | 1.0900 | 1.2000 |
|  | 1.1590 | 1.4500 |
|  | 1.0390 | 1.0950 |
|  | 1.4710 | 1.5920 |
|  | 1.3650 | 1.3390 |
|  | 0.8710 | 1.0070 |
|  | 0.7560 | 1.1120 |
|  | 1.4130 | 2.0940 |
|  | 1.6320 | 1.9530 |
|  | 1.4710 | 0.8360 |
|  | 1.2940 | 1.0080 |
|  | 1.1170 | 1.1120 |
|  | 0.9740 | 0.8560 |
|  | 1.2710 | 1.2810 |
|  | 1.3260 | 1.1180 |
|  | 1.1490 | 0.5420 |
|  | 1.1890 | 0.8050 |
|  | 1.0900 | 0.8960 |
|  | 1.2570 | 0.7960 |
|  | 0.6530 | 1.0190 |
|  | 1.1990 | 0.6230 |
|  | 0.6740 | 0.6060 |
|  | 1.1030 | 1.0120 |
|  | 0.9820 | 0.8120 |
|  | 1.1990 | 0.8120 |
|  | 0.9630 | 0.8690 |
|  | 0.7560 | 1.5310 |
|  | 0.4680 | 1.5110 |
|  | 0.8310 | 0.9120 |
|  | 0.7640 | 1.7120 |
|  | 1.1450 | 0.5420 |
|  | 0.8610 | 0.6850 |
|  | 0.7410 | 0.5600 |
|  | 1.0400 | 0.9060 |
|  | 0.7850 | 0.5060 |
|  | 1.2670 | 0.7210 |
|  | 1.0960 | 0.5740 |
|  | 0.9310 | 0.4530 |
|  | 1.1940 | 0.9070 |
|  | 1.1960 | 0.8120 |
|  | 1.1030 | 0.7120 |
|  | 0.9310 | 1.2460 |
|  | 0.8310 | 0.8380 |
|  | 2.4320 | 0.8120 |
|  | 1.4170 | 0.7550 |
|  | 1.3710 | 0.5540 |
|  | 1.3070 | 0.7550 |
|  | 1.7500 | 0.8070 |
|  | 1.4710 | 1.1740 |
|  | 1.0250 | 0.7550 |
|  | 1.4460 | 0.9000 |
|  | 1.1190 | 0.8440 |
|  | 1.1190 | 0.9070 |
|  | 1.0350 | 1.1120 |
|  | 1.1030 | 1.6830 |
|  | 1.4160 | 0.9470 |
|  | 1.1910 | 0.7030 |
|  | 1.0390 | 1.4690 |
|  | 1.2760 | 0.9470 |
|  | 0.6860 | 1.0250 |
|  | 1.2390 | 0.7790 |
|  | 0.8510 | 1.3730 |
|  | 1.6520 | 1.0010 |
|  | 1.3640 | 1.3460 |
|  | 1.6780 | 0.9470 |
|  | 1.3850 | 0.7030 |
|  | 1.3640 | 1.5810 |
|  | 1.2880 | 1.2610 |
|  | 0.8380 | 0.9440 |
|  | 0.7840 | 0.9440 |
|  | 1.9030 | 0.4720 |
|  | 1.7540 | 0.7080 |
|  | 1.0830 | 1.1690 |
|  | 0.8510 | 1.1820 |
|  | 1.0120 | 0.9950 |
|  | 1.2760 | 0.7750 |
|  | 1.4200 | 0.8510 |
|  | 1.2320 | 0.8510 |
|  | 0.8780 | 0.7080 |
|  | 1.0400 | 0.8970 |
|  | 0.5660 | 0.9200 |
|  | 0.9740 | 0.9470 |
|  | 0.8890 | 1.0250 |
|  | 0.7020 | 1.2230 |
|  | 1.3900 | 0.8210 |
|  | 0.8310 | 1.2680 |
|  | 0.8100 | 0.8020 |
|  | 1.0470 | 1.3390 |
|  | 1.3090 | 1.1120 |
|  | 0.8100 | 1.0580 |
|  | 1.1030 | 1.1120 |
|  | 1.6820 | 1.1260 |
|  | 0.7410 | 0.8470 |
|  | 0.8610 | 1.2610 |
|  | 1.0960 | 0.9950 |
|  | 0.6650 | 1.0580 |
|  | 0.6650 | 0.9600 |
|  | 1.7980 | 1.1040 |
|  | 0.8330 | 0.9470 |
|  | 1.5300 | 1.7270 |
|  | 1.2270 | 0.9470 |
|  | 1.6390 | 1.8910 |
|  | 1.7350 | 1.2210 |
|  | 1.2550 | 1.8240 |
|  | 0.6940 | 1.4180 |
|  | 1.3700 | 0.7250 |
|  | 0.8720 | 1.0410 |
|  | 1.1590 | 1.1550 |
|  | 1.0300 | 1.0470 |
|  | 0.4420 | 0.7170 |
|  | 1.3970 | 1.3230 |
|  | 1.5340 | 1.3740 |
|  | 1.4610 | 1.2600 |
|  | 1.0470 | 1.0250 |
|  | 0.9010 | 1.0000 |
|  | 1.2350 | 1.3370 |
|  | 1.1550 | 1.0220 |
|  | 1.2850 | 0.7460 |
|  | 0.4680 | 0.9500 |
|  | 0.8470 | 1.2000 |
|  | 1.0460 | 0.9730 |
|  | 1.0360 | 1.9150 |
|  | 1.7330 | 1.1260 |
|  | 0.8240 | 1.6210 |
|  | 0.7560 | 1.1690 |
|  | 1.1440 | 0.9470 |
|  | 0.9820 | 0.9470 |
|  | 0.3490 | 0.8970 |
|  | 0.6890 | 1.4500 |
|  | 1.2240 | 0.9470 |
|  | 1.0330 | 1.3730 |
|  | 1.5490 | 0.8690 |
|  | 0.4900 | 0.6140 |
|  | 0.5670 | 1.5510 |
|  | 0.7200 | 0.8100 |
|  | 1.1750 | 0.9170 |
|  | 1.5040 | 1.1040 |
|  | 1.6390 | 1.6830 |
|  | 1.1270 | 0.7900 |
|  | 0.3540 | 0.7030 |
|  | 0.9670 | 0.7860 |
|  | 1.6390 | 0.9600 |
|  | 1.4990 | 1.1260 |
|  | 1.7350 | 0.8650 |
|  | 1.4940 | 1.2000 |
|  | 0.9820 | 0.9470 |
|  | 1.5890 | 1.2000 |
|  | 1.5040 | 0.5990 |
|  | 1.1080 | 1.3730 |
|  | 0.8870 | 1.5810 |
|  | 0.7520 | 0.7120 |
|  | 1.2270 | 0.7750 |
|  | 1.0980 | 0.8650 |
|  | 1.3630 | 0.8470 |
|  | 0.7940 | 1.0220 |
|  | 1.0910 | 1.3580 |
|  | 1.1230 | 1.0250 |
|  | 2.0470 | 1.1040 |
|  | 0.9530 | 1.5510 |
|  | 1.3220 | 1.2430 |
|  | 0.6860 | 0.8970 |
|  | 0.7610 | 1.3910 |
|  | 0.9920 | 0.7420 |
|  | 1.0520 | 1.7320 |
|  | 1.0350 | 1.3190 |
|  | 1.1080 | 1.2820 |
|  | 1.4870 | 0.6950 |
|  | 1.2180 | 0.8400 |
|  | 0.8510 | 1.4310 |
|  | 0.9920 | 0.8110 |
|  | 1.0280 | 0.9830 |
|  | 1.2180 | 1.3370 |
|  | 1.3750 | 0.9330 |
|  | 0.9700 | 1.5290 |
|  | 1.8040 | 1.0590 |
|  | 1.2090 | 0.8790 |
|  | 1.0900 | 1.2320 |
|  | 1.2090 | 1.9780 |
|  | 1.3450 | 1.5010 |
|  | 1.0400 | 1.6740 |
|  | 0.8980 | 1.0990 |
|  | 0.8930 | 0.7930 |
|  | 1.1550 | 0.7930 |
|  | 1.1780 | 0.8460 |
|  | 0.8520 | 1.4070 |
|  | 1.0460 | 1.2780 |
|  | 1.0330 | 0.9830 |
|  | 1.1030 | 1.0260 |
|  | 1.0570 | 1.3120 |
|  | 1.1780 | 1.0860 |
|  | 1.3080 | 1.7500 |
|  | 1.3710 | 1.6740 |
|  | 1.4610 | 1.7690 |
|  | 1.0730 | 1.1800 |
|  | 0.8240 | 0.7930 |
|  | 0.8860 | 0.7490 |
|  | 0.7640 | 1.2320 |
|  | 1.2760 | 1.6710 |
|  | 0.9320 | 0.9730 |
|  | 1.2630 | 1.0450 |
|  | 1.7100 | 1.6740 |
|  | 0.8330 | 1.2590 |
|  | 1.2520 | 0.8110 |
|  | 1.0360 | 0.9880 |
|  | 0.8730 | 1.1840 |
|  | 1.0040 | 1.1950 |
|  | 0.8610 | 0.9770 |
|  | 0.6620 | 0.8760 |
|  | 1.7430 | 0.5940 |
|  | 2.3470 | 0.7880 |
|  | 1.4170 | 1.4220 |
|  | 1.1230 | 1.6990 |
|  | 1.0810 | 1.2650 |
|  | 1.0400 | 1.4070 |
|  | 0.8980 | 1.1360 |
|  | 0.8930 | 1.7310 |
|  | 1.1550 | 1.0710 |
|  | 1.1780 | 0.8760 |
|  | 0.8520 | 0.7780 |
|  | 1.0460 | 1.5470 |
|  | 1.0330 | 1.0140 |
|  | 1.1030 | 0.9010 |
|  | 1.0570 | 0.9520 |
|  | 1.4610 | 1.3550 |
|  | 1.0730 | 0.6950 |
|  | 0.8240 | 0.7360 |
|  | 0.8860 | 1.1340 |
|  | 1.3080 | 0.9350 |
|  | 1.3710 | 1.2360 |
|  | 0.7640 | 1.3840 |
|  | 1.2760 | 0.9460 |
|  | 0.9320 | 0.8200 |
|  | 1.2630 | 1.3210 |
|  | 1.7100 | 1.5880 |
|  | 0.8330 | 1.2020 |
|  | 0.6620 | 0.5660 |
|  | 1.7430 | 1.0750 |
|  | 2.3470 | 1.5150 |
|  | 1.4170 | 2.7060 |
|  | 1.1230 | 1.0700 |
|  | 1.0810 | 0.6920 |
|  | 1.2060 | 0.7580 |
|  | 1.4440 | 1.0260 |
|  | 1.3880 | 1.3970 |
|  | 1.9490 | 1.1210 |
|  | 1.1570 | 1.2430 |
|  | 1.0760 | 1.2000 |
|  | 1.3610 | 1.3900 |
|  | 0.7490 | 1.9880 |
|  | 1.2550 | 1.5000 |
|  | 1.5710 | 1.8910 |
|  | 0.9550 | 1.2930 |
|  | 0.6810 | 1.2780 |
|  | 1.1570 | 0.6590 |
|  | 0.8220 | 1.5040 |
|  | 0.7520 | 0.8900 |
|  | 1.2840 | 1.2780 |
|  | 1.3480 | 1.0990 |
|  | 1.8920 | 0.9680 |
|  | 0.8870 | 1.4880 |
|  | 1.7390 | 1.3900 |
|  | 1.5710 | 1.0260 |
|  | 0.4910 | 1.1210 |
|  | 0.6810 | 0.9880 |
|  | 1.0300 | 0.7090 |
|  | 1.0120 | 0.8790 |
|  | 1.4610 | 0.9060 |
|  | 1.6390 | 0.7090 |
|  | 1.1590 | 0.7930 |
|  | 1.3080 | 1.1200 |
|  | 1.6690 | 2.4600 |
|  | 1.1710 | 1.3120 |
|  | 1.2910 | 1.4070 |
|  | 1.3090 | 1.0860 |
|  | 1.0270 | 1.1960 |
|  | 0.8710 | 1.1880 |
|  | 0.8780 | 1.3120 |
|  | 1.0360 | 0.8460 |
|  | 1.0350 | 0.9060 |
|  | 1.0900 | 0.5560 |
|  | 1.2670 | 0.8850 |
|  | 1.7970 | 1.2820 |
|  | 0.9510 | 1.8700 |
|  | 1.1490 | 1.3760 |
|  | 1.1730 | 0.8850 |
|  | 1.1110 | 0.8850 |
|  | 1.0520 | 1.3760 |
|  | 1.4170 | 0.5010 |
|  | 0.3850 | 1.9880 |
|  | 1.3880 | 1.5260 |
|  | 1.3450 | 1.1840 |
|  | 1.0780 | 0.7490 |
|  | 1.4660 | 0.9680 |
|  | 0.6350 | 0.8340 |
|  | 1.0040 | 0.9730 |
|  | 2.6620 | 1.5090 |
|  | 2.7890 | 0.7550 |
|  | 0.9310 | 1.1610 |
|  | 1.3650 | 0.9170 |
|  | 0.9820 | 0.8970 |
|  | 1.1010 | 0.8360 |
|  | 0.7270 | 0.6720 |
|  | 0.8330 | 0.7250 |
|  | 1.7780 | 1.0930 |
|  | 0.8100 | 1.6830 |
|  | 1.1030 | 0.7460 |
|  | 0.9630 | 0.7420 |
|  | 1.3720 | 0.7860 |
|  | 1.7520 | 1.0250 |
|  | 1.2760 | 0.5280 |
|  | 1.0850 | 0.4720 |
|  | 2.3330 | 0.9440 |
|  | 0.8470 | 1.1120 |
|  | 1.3610 | 0.6290 |
|  | 0.8170 | 1.1900 |
|  | 1.0040 | 0.9470 |
|  | 0.7740 | 0.9570 |
|  | 1.2670 | 0.7460 |
|  | 1.0130 | 0.8970 |
|  | 1.2670 | 1.0550 |
|  | 1.0470 | 0.8790 |
|  | 0.8170 | 1.1120 |
|  | 1.3170 | 0.9850 |
|  | 1.0330 | 1.2800 |
|  | 1.5120 | 1.6590 |
|  | 1.5570 | 0.8790 |
|  | 1.3210 | 1.4500 |
|  | 1.1730 | 1.2710 |
|  | 1.5480 | 1.1040 |
|  | 1.5060 | 1.1610 |
|  | 0.6560 | 1.2580 |
|  | 1.4660 | 0.9470 |
|  | 1.6020 | 1.4840 |
|  | 1.3610 | 1.3850 |
|  | 0.8980 | 0.9700 |
|  | 1.9820 | 0.8680 |
|  | 2.4110 | 1.1090 |
|  | 1.1210 | 1.4490 |
|  | 1.4870 | 1.3290 |
|  | 1.1190 | 2.1520 |
|  | 1.0210 | 1.3450 |
|  | 1.4260 | 1.0660 |
|  | 1.3020 | 1.2560 |
|  | 1.3210 | 1.3290 |
|  | 1.3720 | 1.2270 |
|  | 1.7540 | 1.3290 |
|  | 0.8880 | 1.4440 |
|  | 1.7020 | 1.3850 |
|  | 1.1570 | 1.1450 |
|  | 1.3290 | 1.3240 |
|  | 1.3850 | 1.3850 |
|  | 1.0250 | 1.0250 |
|  | 1.0250 | 1.5040 |
|  | 1.4880 | 1.0470 |
|  | 1.0390 | 1.3830 |
|  | 1.2650 | 1.0090 |
|  | 1.2650 | 0.9510 |
|  | 1.2990 | 1.1700 |
|  | 1.0520 | 0.9810 |
|  | 0.9920 | 0.7740 |
|  | 1.2990 | 1.1100 |
|  | 1.1420 | 1.3830 |
|  | 0.9920 | 1.5670 |
|  | 1.0660 | 0.6380 |
|  | 1.2030 | 1.0690 |
|  | 1.2030 | 0.8570 |
|  | 1.4690 | 1.5040 |
|  | 1.2560 | 1.2810 |
|  | 0.7700 | 0.7440 |
|  | 1.6520 | 0.7520 |
|  | 1.1820 | 1.3160 |
|  | 1.5220 | 0.7440 |
|  | 1.0390 | 1.1750 |
|  | 0.8510 | 1.4890 |
|  | 1.2030 | 1.3830 |
|  | 1.1570 | 1.4310 |
|  | 0.9700 | 1.2940 |
|  | 0.9040 | 1.1500 |
|  | 0.9920 | 1.3160 |
|  | 1.0900 | 0.7520 |
|  | 1.0390 | 1.1450 |
|  | 1.0900 | 1.3830 |
|  | 0.9700 | 1.3870 |
|  | 1.6260 | 0.7440 |
|  | 0.9510 | 1.1890 |
|  | 1.0760 | 1.3830 |
|  | 0.7840 | 0.9150 |
|  | 0.9920 | 0.9150 |
|  | 0.4810 | 0.7740 |
|  | 1.2930 | 0.9150 |
|  | 0.8850 | 1.5490 |
|  | 0.9480 | 1.1890 |
|  | 0.9260 | 0.7740 |
|  | 0.9550 | 1.3540 |
|  | 0.8200 | 1.0030 |
|  | 0.7010 | 1.0030 |
|  | 1.1590 | 0.8310 |
|  | 1.3430 | 1.3620 |
|  | 1.0370 | 0.9810 |
|  | 0.7610 | 1.0640 |
|  | 0.8720 | 0.8570 |
|  | 0.7760 | 1.2560 |
|  | 0.6460 | 0.7840 |
|  | 1.1910 | 0.9400 |
|  | 0.7760 | 0.9510 |
|  | 1.3630 | 0.9960 |
|  | 0.6810 | 0.7320 |
|  | 1.4660 | 1.1820 |
|  | 1.0120 | 1.2560 |
|  | 1.0120 | 0.9040 |
|  | 0.9700 | 0.8510 |
|  | 0.8510 | 0.9510 |
|  | 1.3750 | 1.3720 |
|  | 0.8420 | 0.9040 |
|  | 1.3330 | 1.3020 |
|  | 1.0030 | 0.9510 |
|  | 1.4270 | 0.8420 |
|  | 0.9510 | 0.7610 |
|  | 0.7670 | 0.6480 |
|  | 0.9090 | 1.0660 |
|  | 1.0640 | 1.1820 |
|  | 0.8770 | 1.4440 |
|  | 0.6200 | 1.1450 |
|  | 1.1310 | 1.2090 |
|  | 1.1890 | 1.2650 |
|  | 1.1450 | 0.8550 |
|  | 1.0850 | 1.2960 |
|  | 0.6730 | 0.8030 |
|  | 0.7740 | 0.9400 |
|  | 0.7440 | 1.2390 |
|  | 0.6810 | 1.3720 |
|  | 1.1310 | 0.9510 |
|  | 1.1450 | 0.7660 |
|  | 1.2180 | 0.8550 |
|  | 0.8030 | 0.9160 |
|  | 1.2180 | 0.8760 |
|  | 1.1940 | 1.1850 |
|  | 1.4490 | 0.9960 |
|  | 1.2560 | 1.1820 |
|  | 0.5380 | 0.7020 |
|  | 1.0250 | 1.1820 |
|  | 1.1060 | 1.1850 |
|  | 0.9960 | 0.8760 |
|  | 1.2560 | 0.8760 |
|  | 0.9510 | 1.0830 |
|  | 0.8550 | 1.0280 |
|  | 0.6480 | 1.2270 |
|  | 1.3750 | 1.6520 |
|  | 1.1940 | 1.0660 |
|  | 0.9700 | 0.9200 |
|  | 1.8410 | 1.3640 |
|  | 1.2760 | 0.7610 |
|  | 1.2030 | 0.7840 |
|  | 0.9920 | 0.6860 |
|  | 0.9200 | 1.2030 |
|  | 0.6020 | 0.9700 |
|  | 1.1450 | 1.0660 |
|  | 0.6860 | 0.9920 |
|  | 0.8030 | 0.7800 |
|  | 1.0660 | 1.4160 |
|  | 1.7870 | 1.0760 |
|  | 0.7490 | 0.9200 |
|  | 0.8200 | 0.9040 |
|  | 0.7490 | 0.7270 |
|  | 2.1280 | 0.8510 |
|  | 1.1590 | 1.1570 |
|  | 1.0890 | 0.8420 |
|  | 1.0120 | 1.4460 |
|  | 0.8870 | 1.0250 |
|  | 1.3700 | 0.9200 |
|  | 0.8850 | 0.6650 |
|  | 0.5860 | 1.3290 |
|  | 1.1890 | 1.0520 |
|  | 1.1650 | 0.7610 |
|  | 1.2550 | 0.8030 |
|  | 0.7610 | 0.8070 |
|  | 1.3080 | 1.1820 |
|  | 1.1160 | 1.2090 |
|  | 0.8850 | 0.7840 |
|  | 0.9550 | 1.0270 |
|  | 0.8280 | 1.6950 |
|  | 1.6060 | 1.1350 |
|  | 0.8280 | 0.8510 |
|  | 1.3000 | 0.8760 |
|  | 1.3000 | 0.6020 |
|  | 0.6840 | 0.9510 |
|  | 1.1060 | 0.9310 |
|  | 1.3640 | 0.9320 |
|  | 0.7020 | 1.1110 |
|  | 0.8070 | 0.6210 |
|  | 0.8510 | 0.6210 |
|  | 0.9510 | 0.8100 |
|  | 1.9570 | 0.9510 |
|  | 0.9200 | 0.9820 |
|  | 0.6020 | 1.0700 |
|  | 1.6430 | 0.9510 |
|  | 1.3290 | 0.9400 |
|  | 1.1940 | 0.8860 |
|  | 1.4540 | 0.9510 |
|  | 1.3290 | 1.0810 |
|  | 0.8880 | 1.2760 |
|  | 0.9510 | 1.0130 |
|  | 1.0660 | 0.8100 |
|  | 1.0660 | 0.6580 |
|  | 1.5080 | 0.8310 |
|  | 1.5410 | 0.9270 |
|  | 1.3720 | 1.1330 |
|  | 1.5080 | 0.9820 |
|  | 1.2960 | 0.8710 |
|  | 1.3450 | 0.8710 |
|  | 0.7270 | 0.6210 |
|  | 0.8380 | 1.1230 |
|  | 1.1570 | 0.8730 |
|  | 1.5340 | 0.8730 |
|  | 1.4490 | 1.0010 |
|  | 1.1060 | 0.8730 |
|  | 0.9400 | 0.7760 |
|  | 0.7700 | 0.7240 |
|  | 1.5320 | 1.1160 |
|  | 1.0900 | 0.9160 |
|  | 1.0760 | 1.1080 |
|  | 1.1450 | 0.7940 |
|  | 1.1820 | 0.8200 |
|  | 1.0250 | 1.2040 |
|  | 0.8420 | 0.5490 |
|  | 0.9040 | 1.2040 |
|  | 1.0250 | 1.0370 |
|  | 1.2960 | 0.7240 |
|  | 1.4540 | 1.1330 |
|  | 0.8380 | 1.0760 |
|  | 0.8880 | 0.5490 |
|  | 0.9700 | 0.9670 |
|  | 0.9960 | 0.7010 |
|  | 1.2270 | 1.0390 |
|  | 1.1450 | 1.0390 |
|  | 1.1060 | 1.1890 |
|  | 0.5380 | 1.2180 |
|  | 1.1090 | 1.0700 |
|  | 0.7660 | 0.8720 |
|  | 1.0520 | 1.5300 |
|  | 1.2990 | 1.5550 |
|  | 1.0250 | 1.4870 |
|  | 0.8880 | 1.0050 |
|  | 1.2650 | 1.9820 |
|  | 1.1060 | 1.3430 |
|  | 0.8550 | 1.6850 |
|  | 0.8550 | 1.1570 |
|  | 1.1090 | 1.0630 |
|  | 1.0660 | 1.4570 |
|  | 1.0250 | 1.1590 |
|  | 1.2880 | 1.3970 |
|  | 0.7770 | 1.0390 |
|  | 0.9160 | 1.1910 |
|  | 0.6700 | 1.0650 |
|  | 1.0890 | 0.7330 |
|  | 0.7760 | 1.4730 |
|  | 0.7610 | 1.6420 |
|  | 0.7940 | 1.2270 |
|  | 0.9630 | 1.0670 |
|  | 0.7010 | 1.1000 |
|  | 1.0980 | 1.0650 |
|  | 0.7970 | 1.1330 |
|  | 1.0230 | 1.0210 |
|  | 0.7520 | 1.0760 |
|  | 0.7490 | 1.8830 |
|  | 0.8850 | 1.0210 |
|  | 1.0050 | 0.9630 |
|  | 1.3780 | 1.0630 |
|  | 0.7240 | 0.7940 |
|  | 0.9080 | 0.8850 |
|  | 1.3510 | 1.0760 |
|  | 1.6240 | 1.2120 |
|  | 0.9630 | 1.0050 |
|  | 0.6130 | 1.1570 |
|  | 1.1590 | 1.3770 |
|  | 1.3560 | 0.8530 |
|  | 1.4020 | 1.0120 |
|  | 1.0120 | 0.7940 |
|  | 0.9670 | 0.9480 |
|  | 1.2270 | 1.0570 |
|  | 0.5450 | 0.9480 |
|  | 1.1730 | 1.2840 |
|  | 1.1200 | 1.2840 |
|  | 0.9160 | 1.1080 |
|  | 0.8380 | 0.6810 |
|  | 1.0170 | 0.9910 |
|  | 0.6760 | 0.6810 |
|  | 1.7560 | 1.1000 |
|  | 0.8320 | 1.0210 |
|  | 0.6160 | 1.6580 |
|  | 1.2950 | 1.4030 |
|  | 0.8590 | 0.8870 |
|  | 0.8570 | 1.7020 |
|  | 1.0390 | 1.0370 |
|  | 0.7790 | 1.0050 |
|  | 0.8260 | 1.2500 |
|  | 1.1720 | 1.2720 |
|  | 0.7710 | 0.8850 |
|  | 0.9790 | 1.1890 |
|  | 0.8820 | 1.1000 |
|  | 0.8940 | 1.4030 |
|  | 0.7720 | 0.7610 |
|  | 0.9740 | 1.0300 |
|  | 1.2700 | 1.7230 |
|  | 1.4990 | 1.1410 |
|  | 0.9740 | 1.1650 |
|  | 0.6650 | 1.4440 |
|  | 0.5250 | 1.2250 |
|  | 0.6240 | 1.1650 |
|  | 0.7720 | 0.6810 |
|  | 0.9590 | 0.9260 |
|  | 1.1090 | 1.5040 |
|  | 0.9820 | 1.3220 |
|  | 1.1570 | 1.2270 |
|  | 1.1000 | 1.0210 |
|  | 0.5450 | 1.0250 |
|  | 1.2040 | 1.2090 |
|  | 1.1160 | 1.0250 |
|  | 0.9820 | 1.2790 |
|  | 1.2230 | 1.2030 |
|  | 1.0630 | 1.1910 |
|  | 0.7110 | 0.6810 |
|  | 0.9630 | 1.3640 |
|  | 1.3090 | 1.1090 |
|  | 1.1630 | 1.5050 |
|  | 1.1910 | 1.4460 |
|  | 0.6160 | 2.2190 |
|  | 1.1650 | 1.4490 |
|  | 0.7760 | 1.1940 |
|  | 1.2790 | 1.3610 |
|  | 0.7360 | 0.9360 |
|  | 1.6560 | 1.1910 |
|  | 0.9750 | 1.0210 |
|  | 1.3000 | 0.7020 |
|  | 0.7330 | 1.5340 |
|  | 0.8610 | 1.2270 |
|  | 1.0230 | 0.9700 |
|  | 0.9630 | 1.4540 |
|  | 1.3680 | 0.9360 |
|  | 1.2330 | 1.3550 |
|  | 0.7610 | 1.5140 |
|  | 0.4760 | 1.6080 |
|  | 0.5450 | 1.9880 |
|  | 0.8200 | 0.8720 |
|  | 0.8220 | 1.7830 |
|  | 0.8950 | 1.0650 |
|  | 0.7520 | 0.7650 |
|  | 1.1330 | 1.1080 |
|  | 1.0760 | 1.1640 |
|  | 1.2180 | 1.2360 |
|  | 0.8950 | 1.2230 |
|  | 0.8950 | 1.3950 |
|  | 1.2180 | 1.2760 |
|  | 0.7010 | 1.0250 |
|  | 1.0760 | 1.0410 |
|  | 1.1330 | 1.1890 |
|  | 1.1550 | 1.1540 |
|  | 0.7700 | 1.7620 |
|  | 1.1080 | 1.1630 |
|  | 1.4020 | 1.3310 |
|  | 0.7110 | 1.3900 |
|  | 1.1330 | 1.0210 |
|  | 0.7240 | 1.6630 |
|  | 0.9630 | 1.2200 |
|  | 0.9260 | 1.7400 |
|  | 0.9160 | 1.3620 |
|  | 0.6840 | 1.5250 |
|  | 1.0630 | 1.1830 |
|  | 0.9260 | 1.2230 |
|  | 0.9130 | 1.5250 |
|  | 1.0390 | 1.0920 |
|  | 0.7940 | 1.4370 |
|  | 1.1000 | 1.7310 |
|  | 0.8660 | 1.0560 |
|  | 1.3080 | 1.1550 |
|  | 0.6810 | 0.9560 |
|  | 0.8200 | 0.9750 |
|  | 1.1080 | 1.0600 |
|  | 1.3080 | 1.2370 |
|  | 0.5180 | 1.3970 |
|  | 0.9460 | 1.5340 |
|  | 1.0300 | 0.9290 |
|  | 1.0370 | 1.1170 |
|  | 1.5830 | 1.7570 |
|  | 1.1000 | 1.6940 |
|  | 0.7970 | 1.2090 |
|  | 0.9750 | 1.0370 |
|  | 0.9130 | 0.8170 |
|  | 1.3560 | 2.1320 |
|  | 1.2840 | 2.1020 |
|  | 1.2760 | 1.6810 |
|  | 0.5490 | 2.9730 |
|  | 0.8310 | 0.7520 |
|  | 1.0210 | 0.7110 |
|  | 1.3240 | 0.6860 |
|  | 0.6860 | 0.9700 |
|  | 1.1940 | 0.8510 |
|  | 0.6020 | 0.7610 |
|  | 1.5900 | 1.1090 |
|  | 0.7020 | 1.0390 |
|  | 0.8760 | 0.7840 |
|  | 0.7840 | 0.4960 |
|  | 0.8380 | 1.2550 |
|  | 1.1090 | 0.9750 |
|  | 1.2650 | 0.8610 |
|  | 1.0250 | 1.5640 |
|  | 0.7840 | 0.9550 |
|  | 0.6860 | 0.9550 |
|  | 1.1090 | 1.0230 |
|  | 0.8380 | 0.7840 |
|  | 0.9920 | 0.6810 |
|  | 0.4580 | 0.6420 |
|  | 0.8510 | 1.0210 |
|  | 1.1420 | 0.6020 |
|  | 1.3240 | 0.6810 |
|  | 0.9510 | 0.8760 |
|  | 0.8510 | 0.7000 |
|  | 0.8760 | 0.7270 |
|  | 1.5220 | 0.5710 |
|  | 1.1060 | 1.1060 |
|  | 1.1350 | 0.9920 |
|  | 0.7700 | 0.4580 |
|  | 1.2500 | 0.7840 |
|  | 0.8350 | 0.8510 |
|  | 0.9550 | 0.8760 |
|  | 0.6840 | 0.8510 |
|  | 0.9530 | 1.3290 |
|  | 1.4610 | 1.6340 |
|  | 1.2550 | 0.6650 |
|  | 0.7360 | 0.9400 |
|  | 0.9630 | 1.4160 |
|  | 0.6810 | 0.6860 |
|  | 0.7610 | 1.7230 |
|  | 1.6140 | 0.9160 |
|  | 1.1590 | 1.3290 |
|  | 0.8310 | 0.9040 |
|  | 1.3700 | 1.1190 |
|  | 0.7610 | 0.7840 |
|  | 1.2930 | 0.8510 |
|  | 0.7330 | 1.6890 |
|  | 0.7240 | 1.3320 |
|  | 1.1000 | 0.9510 |
|  | 0.6280 | 1.4080 |
|  | 1.1890 | 0.8510 |
|  | 1.2840 | 0.6820 |
|  | 0.7010 | 1.5300 |
|  | 1.6390 | 1.5600 |
|  | 1.1590 | 1.1060 |
|  | 0.9260 | 0.9400 |
|  | 1.4610 | 1.2330 |
|  | 1.2270 | 0.7490 |
|  | 1.0470 | 1.5520 |
|  | 0.9820 | 0.8280 |
|  | 0.5790 | 0.8280 |
|  | 0.7580 | 1.3630 |
|  | 0.7020 | 1.1590 |
|  | 1.1450 | 1.0210 |
|  | 0.6650 | 0.8420 |
|  | 0.7840 | 1.5550 |
|  | 1.1570 | 0.8870 |
|  | 0.8420 | 1.0050 |
|  | 1.2090 | 0.9260 |
|  | 1.2030 | 0.8850 |
|  | 1.1570 | 1.1740 |
|  | 0.8420 | 1.0760 |
|  | 0.9510 | 1.1410 |
|  | 0.8760 | 1.1940 |
|  | 0.9510 | 1.1090 |
|  | 1.3950 | 1.3610 |
|  | 0.8510 | 1.3640 |
|  | 0.9160 | 1.3720 |
|  | 0.9700 | 1.5830 |
|  | 1.9120 | 1.2560 |
|  | 1.2650 | 1.5340 |
|  | 0.8550 | 1.3210 |
|  | 0.9700 | 0.7320 |
|  | 0.7700 | 0.8880 |
|  | 0.8510 | 1.1190 |
|  | 1.2990 | 1.9400 |
|  | 1.2030 | 1.9400 |
|  | 0.8030 | 1.3020 |
|  | 0.7320 | 1.4640 |
|  | 1.1060 | 1.3720 |
|  | 0.9160 | 1.0390 |
|  | 0.9510 | 1.3450 |
|  | 0.6810 | 0.9920 |
|  | 1.2850 | 1.3210 |
|  | 0.5890 | 1.8740 |
|  | 0.5890 | 1.7870 |
|  | 0.6650 | 1.3640 |
|  | 1.5480 | 1.7890 |
|  | 0.9650 | 0.7610 |
|  | 0.9820 | 0.9700 |
|  | 0.9060 | 1.4640 |
|  | 1.0950 | 1.7890 |
|  | 0.6930 | 1.1090 |
|  | 0.6210 | 1.7100 |
|  | 0.7330 | 0.9510 |
|  | 1.7090 | 0.7700 |
|  | 0.8610 | 0.8680 |
|  | 1.1170 | 0.7840 |
|  | 0.8730 | 1.1420 |
|  | 1.0460 | 1.0250 |
|  | 0.9010 | 1.4490 |
|  | 0.9510 | 1.1910 |
|  | 1.2700 | 1.1940 |
|  | 1.1650 | 1.6260 |
|  | 1.2910 | 1.1060 |
|  | 0.5170 | 1.5340 |
|  | 1.0040 | 1.5340 |
|  | 0.5450 | 1.7040 |
|  | 1.2030 | 1.0250 |
|  | 0.8780 | 0.8510 |
|  | 1.0120 | 1.5410 |
|  | 0.7360 | 1.9590 |
|  | 1.8120 | 1.0210 |
|  | 1.2500 | 1.0250 |
|  | 0.8850 | 1.1910 |
|  | 1.0980 | 1.3610 |
|  | 1.2180 | 1.1060 |
|  | 2.2390 | 1.3640 |
|  | 0.7760 | 1.7890 |
|  | 0.7700 | 1.2760 |
|  | 1.3770 | 1.5640 |
|  | 0.9630 | 1.2180 |
|  | 1.1550 | 1.2030 |
|  | 1.5710 | 1.2650 |
|  | 1.4160 | 1.4490 |
|  | 0.6160 | 1.5320 |
|  | 1.4990 | 1.3450 |
|  | 1.0120 | 1.3830 |
|  | 0.8310 | 1.7700 |
|  | 0.8870 | 1.5950 |
|  | 0.7760 | 1.9140 |
|  | 0.7760 | 1.3870 |
|  | 1.3610 | 1.7020 |
|  | 0.8850 | 1.8110 |
|  | 0.2500 | 1.4930 |
|  | 0.5450 | 1.1700 |
|  | 0.4710 | 1.6820 |
|  | 1.0060 | 1.7700 |
|  | 1.0490 | 1.5990 |
|  | 0.6210 | 1.6090 |
|  | 0.9940 | 1.2400 |
|  | 1.2540 | 1.3870 |
|  | 0.4930 | 1.5700 |
|  | 1.0040 | 1.3450 |
|  | 1.0010 | 1.5950 |
|  | 0.3960 | 1.5040 |
|  | 1.3880 | 1.3620 |
|  | 1.2090 | 0.8420 |
|  | 1.9670 | 1.3290 |
|  | 0.5450 | 1.3290 |
|  | 0.7410 | 1.2650 |
|  | 0.9320 | 1.3850 |
|  | 0.8980 | 1.6890 |
|  | 0.7310 | 1.2270 |
|  | 0.8780 | 1.3290 |
|  | 0.8190 | 0.9920 |
|  | 1.0950 | 0.9200 |
|  | 0.9510 | 0.9510 |
|  | 0.9630 | 1.3450 |
|  | 1.0780 | 1.3850 |
|  | 1.1170 | 1.1090 |
|  | 1.2540 | 1.0630 |
|  | 0.6620 | 1.2550 |
|  | 0.8240 | 1.8390 |
|  |  | 2.1180 |
|  |  | 1.0630 |
|  |  | 0.9040 |
|  |  | 1.1750 |
|  |  | 0.8720 |
|  |  | 1.0980 |
|  |  | 1.1410 |
|  |  | 1.1570 |
|  |  | 1.1700 |
|  |  | 1.5220 |
|  |  | 1.1570 |
|  |  | 1.7070 |
|  |  | 1.1570 |
|  |  | 1.5220 |
|  |  | 1.3560 |
|  |  | 0.8200 |
|  |  | 1.1710 |
|  |  | 0.7970 |
|  |  | 0.8170 |
|  |  | 1.7870 |
|  |  | 1.3220 |
|  |  | 1.5710 |
|  |  | 0.9550 |
|  |  | 1.1890 |
|  |  | 1.2950 |
|  |  | 0.9910 |
|  |  | 1.1650 |
|  |  | 0.8610 |
|  |  | 1.7810 |
|  |  | 1.3700 |
|  |  | 1.4030 |
|  |  | 1.2790 |
|  |  | 1.3080 |
|  |  | 0.7940 |
|  |  | 1.1570 |
|  |  | 1.2180 |
|  |  | 2.0110 |
|  |  | 1.2910 |
|  |  | 0.8870 |
|  |  | 1.1910 |
|  |  | 1.0300 |
|  |  | 1.0120 |
|  |  | 0.9260 |
|  |  | 0.5610 |
|  |  | 0.9670 |
|  |  | 1.7350 |
|  |  | 1.0570 |
|  |  | 1.3610 |
|  |  | 1.3780 |
|  |  | 0.7970 |
|  |  | 0.6090 |
|  |  | 1.0370 |
|  |  | 0.9550 |
|  |  | 1.4360 |
|  |  | 1.4560 |
|  |  | 1.0210 |
|  |  | 1.1910 |
|  |  | 0.5110 |
|  |  | 1.2180 |
|  |  | 1.1940 |
|  |  | 1.5690 |
|  |  | 1.2650 |
|  |  | 1.5710 |
|  |  | 1.6020 |
|  |  | 1.6560 |
|  |  | 1.6450 |
|  |  | 1.0210 |
|  |  | 1.5300 |
|  |  | 1.7350 |
|  |  | 0.6280 |
|  |  | 1.2910 |
|  |  | 1.2270 |
|  |  | 1.3780 |
|  |  | 1.8730 |
|  |  | 1.1820 |
|  |  | 1.2090 |
|  |  | 1.2270 |
|  |  | 1.5050 |
|  |  | 0.7330 |
|  |  | 0.9750 |
|  |  | 1.0630 |
|  |  | 0.8310 |
|  |  | 0.9670 |
|  |  | 1.1650 |
|  |  | 0.9260 |
|  |  | 1.5220 |
|  |  | 1.3630 |
|  |  | 0.8950 |
|  |  | 0.8200 |
|  |  | 0.9130 |
|  |  | 0.8870 |
|  |  | 0.7110 |
|  |  | 0.7520 |
|  |  | 1.2790 |
|  |  | 1.0210 |
|  |  | 1.7180 |
|  |  | 0.7490 |
|  |  | 0.8310 |
|  |  | 0.7940 |
|  |  | 0.7940 |
|  |  | 0.8800 |
|  |  | 0.9160 |
|  |  | 0.9630 |
|  |  | 0.9670 |
|  |  | 0.8660 |
|  |  | 1.7350 |
|  |  | 1.3770 |
|  |  | 0.9630 |
|  |  | 1.7000 |
|  |  | 1.2270 |
|  |  | 1.2650 |
|  |  | 1.2130 |
|  |  | 1.0210 |
|  |  | 1.8740 |
|  |  | 1.5340 |
|  |  | 1.2030 |
|  |  | 1.6650 |
|  |  | 1.8600 |
|  |  | 1.6200 |
|  |  | 1.6230 |
|  |  | 1.0690 |
|  |  | 1.0850 |
|  |  | 1.0530 |
|  |  | 1.0850 |
|  |  | 1.1890 |
|  |  | 1.2810 |
|  |  | 1.2940 |
|  |  | 1.0640 |
|  |  | 1.7050 |
|  |  | 1.4930 |
|  |  | 0.9150 |
|  |  | 0.7670 |
|  |  | 0.6810 |
|  |  | 0.7440 |
|  |  | 1.2760 |
|  |  | 1.4930 |
|  |  | 1.8210 |
|  |  | 1.5040 |
|  |  | 1.7150 |
|  |  | 1.2450 |
|  |  | 1.7050 |
|  |  | 1.9140 |
|  |  | 1.2450 |
|  |  | 2.5030 |
|  |  | 1.6270 |
|  |  | 1.7540 |
|  |  | 1.8570 |
|  |  | 1.9610 |
|  |  | 1.5230 |
|  |  | 1.7830 |
|  |  | 1.5490 |
|  |  | 1.0690 |
|  |  | 0.9150 |
|  |  | 1.2130 |
|  |  | 1.3540 |
|  |  | 1.3870 |
|  |  | 1.3160 |
|  |  | 1.1890 |
|  |  | 1.8300 |
|  |  | 0.8070 |
|  |  | 1.3850 |
|  |  | 1.2560 |
|  |  | 0.8880 |
|  |  | 0.9630 |
|  |  | 1.2650 |
|  |  | 1.1910 |
|  |  | 0.7840 |
|  |  | 0.9920 |
|  |  | 1.1450 |
|  |  | 1.8720 |
|  |  | 1.1420 |
|  |  | 1.1940 |
|  |  | 0.9700 |
|  |  | 1.5150 |
|  |  | 0.8880 |
|  |  | 0.7700 |
|  |  | 1.2270 |
|  |  | 1.6260 |
|  |  | 1.2770 |
|  |  | 1.6260 |
|  |  | 1.3850 |
|  |  | 1.6340 |
|  |  | 1.4540 |
|  |  | 1.1450 |
|  |  | 1.3720 |
|  |  | 1.0250 |
|  |  | 0.7610 |
|  |  | 1.0900 |
|  |  | 1.4640 |
|  |  | 1.6890 |
|  |  | 0.9510 |
|  |  | 0.7840 |
|  |  | 1.2990 |
|  |  | 2.1130 |
|  |  | 1.7460 |
|  |  | 1.5830 |
|  |  | 0.7840 |
|  |  | 1.1940 |
|  |  | 1.1910 |
|  |  | 1.4860 |
|  |  | 1.9200 |
|  |  | 1.2650 |
|  |  | 1.2560 |
|  |  | 1.7540 |
|  |  | 0.7610 |
|  |  | 0.8380 |
|  |  | 1.0250 |
|  |  | 1.5690 |
|  |  | 1.2390 |
|  |  | 1.2180 |
|  |  | 1.2180 |
|  |  | 0.9160 |
|  |  | 1.4540 |
|  |  | 1.7310 |
|  |  | 1.5700 |
|  |  | 1.2130 |
|  |  | 1.7150 |
|  |  | 1.2130 |
|  |  | 1.6650 |
|  |  | 1.5810 |
|  |  | 1.4930 |
|  |  | 2.0240 |
|  |  | 1.0640 |
|  |  | 1.4810 |
|  |  | 1.2810 |
|  |  | 1.8360 |
|  |  | 0.7740 |
|  |  | 1.2400 |
|  |  | 1.2270 |
|  |  | 1.6760 |
|  |  | 0.9200 |
|  |  | 1.2650 |
|  |  | 1.4260 |
|  |  | 1.7460 |
|  |  | 0.9510 |
|  |  | 1.4690 |
|  |  | 1.2790 |
|  |  | 0.7660 |
|  |  | 1.1190 |
|  |  | 0.7700 |
|  |  | 1.4460 |
|  |  | 1.7040 |
|  |  | 1.1090 |
|  |  | 0.8550 |
|  |  | 1.0900 |
|  |  | 0.9700 |
|  |  | 0.7700 |
|  |  | 1.1570 |
|  |  | 0.7700 |
|  |  | 1.0280 |
|  |  | 1.0390 |
|  |  | 1.1940 |
|  |  | 1.6370 |
|  |  | 0.8070 |
|  |  | 1.5700 |
|  |  | 0.7670 |
|  |  | 1.8170 |
|  |  | 1.5230 |
|  |  | 1.6610 |
|  |  | 1.6820 |
|  |  | 1.3450 |
|  |  | 0.7670 |
|  |  | 1.6230 |
|  |  | 1.1500 |
|  |  | 1.4470 |
|  |  | 1.2400 |
|  |  | 1.8890 |
|  |  | 1.2810 |
|  |  | 1.8570 |
|  |  | 1.8570 |
|  |  | 1.3210 |
|  |  | 1.2980 |
|  |  | 0.9360 |
|  |  | 1.2390 |
|  |  | 1.3110 |
|  |  | 1.0690 |
|  |  | 0.9570 |
|  |  | 1.6510 |
|  |  | 1.2130 |
|  |  | 1.3960 |
|  |  | 1.1450 |
|  |  | 1.2630 |
|  |  | 1.1640 |
|  |  | 2.1810 |
|  |  | 0.8780 |
|  |  | 0.8190 |
|  |  | 1.5570 |
|  |  | 1.1330 |
|  |  | 0.8190 |
|  |  | 1.0010 |
|  |  | 1.2760 |
|  |  | 1.1170 |
|  |  | 0.7410 |
|  |  | 1.5260 |
|  |  | 0.9740 |
|  |  | 1.0000 |
|  |  | 0.9310 |
|  |  | 0.8930 |
|  |  | 1.4200 |
|  |  | 1.1780 |
|  |  | 1.4000 |
|  |  | 1.8000 |
|  |  | 0.9270 |
|  |  | 1.8640 |
|  |  | 1.5260 |
|  |  | 0.8040 |
|  |  | 1.9820 |
|  |  | 1.3480 |
|  |  | 1.1550 |
|  |  | 1.2090 |
|  |  | 0.7800 |
|  |  | 1.0230 |
|  |  | 1.4660 |
|  |  | 1.3650 |
|  |  | 1.2910 |
|  |  | 1.5020 |
|  |  | 1.6780 |
|  |  | 0.6560 |
|  |  | 1.6600 |
|  |  | 1.0850 |
|  |  | 0.8710 |
|  |  | 0.9940 |
|  |  | 0.9820 |
|  |  | 1.2570 |
|  |  | 1.3610 |
|  |  | 1.8000 |
|  |  | 0.8190 |
|  |  | 0.8330 |
|  |  | 1.2444 |
|  |  | 0.5990 |
|  |  | 0.9800 |
|  |  | 0.9270 |
|  |  | 1.2240 |
|  |  | 1.5420 |
|  |  | 1.4380 |
|  |  | 1.6600 |
|  |  | 1.3110 |
|  |  | 1.1500 |
|  |  | 1.6890 |
|  |  | 1.4470 |
|  |  | 1.0170 |
|  |  | 1.0330 |
|  |  | 1.1230 |
|  |  | 1.3690 |
|  |  | 1.2910 |
|  |  | 1.0010 |
|  |  | 1.3160 |
|  |  | 1.2750 |
|  |  | 0.9530 |
|  |  | 1.2420 |
|  |  | 1.5590 |
|  |  | 1.1940 |
|  |  | 0.9820 |
|  |  | 1.2180 |
|  |  | 0.8170 |
|  |  | 1.1650 |
|  |  | 1.0360 |
|  |  | 1.9070 |
|  |  | 0.8190 |
|  |  | 1.0210 |
|  |  | 1.6170 |
|  |  | 0.9360 |
|  |  | 1.2390 |
|  |  | 1.6170 |
|  |  | 1.1110 |
|  |  | 1.2650 |
|  |  | 1.1090 |
|  |  | 1.1540 |
|  |  | 1.2990 |
|  |  | 0.9260 |
|  |  | 1.0330 |
|  |  | 1.1990 |
|  |  | 0.9650 |
|  |  | 1.0780 |
|  |  | 1.1940 |
|  |  | 1.5480 |
|  |  | 1.5420 |
|  |  | 0.9700 |
|  |  | 0.9860 |
|  |  | 1.2420 |
|  |  | 1.2540 |
|  |  | 1.0460 |
|  |  | 1.6250 |
|  |  | 1.1330 |
|  |  | 0.9750 |
|  |  | 1.2510 |
|  |  | 1.2500 |
|  |  | 1.4570 |
|  |  | 1.1230 |
|  |  | 0.9460 |
|  |  | 1.0390 |
|  |  | 0.6810 |
|  |  | 0.8530 |
|  |  | 0.7940 |
|  |  | 0.6090 |
|  |  | 1.0050 |
|  |  | 0.9130 |
|  |  | 0.9910 |
|  |  | 1.0050 |
|  |  | 0.7240 |
|  |  | 0.8530 |
|  |  | 1.0980 |
|  |  | 0.8310 |
|  |  | 0.8510 |
|  |  | 0.7840 |
|  |  | 1.0760 |
|  |  | 1.0210 |
|  |  | 1.2760 |
|  |  | 1.5340 |
|  |  | 1.3450 |
|  |  | 0.7270 |
|  |  | 0.6860 |
|  |  | 1.2990 |
|  |  | 0.9160 |
|  |  | 1.1350 |
|  |  | 1.3640 |
|  |  | 1.2790 |
|  |  | 1.0250 |
|  |  | 0.8550 |
|  |  | 0.8030 |
|  |  | 1.2650 |
|  |  | 1.0520 |
|  |  | 1.2560 |
|  |  | 1.4030 |
|  |  | 0.9700 |
|  |  | 1.8050 |
|  |  | 1.1850 |
|  |  | 0.9700 |
|  |  | 0.9700 |
|  |  | 1.1350 |
|  |  | 1.0350 |
|  |  | 1.0760 |
|  |  | 0.9920 |
|  |  | 1.0250 |
|  |  | 0.7320 |
|  |  | 1.4490 |
|  |  | 1.0250 |
|  |  | 0.5960 |
|  |  | 1.2270 |
|  |  | 0.9400 |
|  |  | 0.9920 |
|  |  | 0.8680 |
|  |  | 0.9510 |
|  |  | 0.8680 |
|  |  | 0.7610 |
|  |  | 1.3020 |
|  |  | 0.7700 |
|  |  | 0.7700 |
|  |  | 1.1060 |
|  |  | 1.2030 |
|  |  | 0.9360 |
|  |  | 0.9360 |
|  |  | 0.8070 |
|  |  | 1.2030 |
|  |  | 0.8030 |
|  |  | 0.8510 |
|  |  | 1.9030 |
|  |  | 1.0640 |
|  |  | 1.2260 |
|  |  | 1.5560 |
|  |  | 1.4680 |
|  |  | 0.9310 |
|  |  | 0.7980 |
|  |  | 1.2260 |
|  |  | 1.0640 |
|  |  | 0.8090 |
|  |  | 1.0720 |
|  |  | 1.8470 |
|  |  | 1.6820 |
|  |  | 1.5950 |
|  |  | 1.0720 |
|  |  | 0.9400 |
|  |  | 1.4860 |
|  |  | 1.0380 |
|  |  | 1.2260 |
|  |  | 0.9310 |
|  |  | 1.3990 |
|  |  | 0.7670 |
|  |  | 1.1890 |
|  |  | 1.4470 |
|  |  | 0.9510 |
|  |  | 1.4930 |
|  |  | 1.4190 |
|  |  | 0.6470 |
|  |  | 0.9090 |
|  |  | 1.0470 |
|  |  | 1.0640 |
|  |  | 0.9810 |
|  |  | 0.9090 |
|  |  | 0.9090 |
|  |  | 0.8510 |
|  |  | 1.1500 |
|  |  | 1.1450 |
|  |  | 1.2810 |
|  |  | 1.0850 |
|  |  | 1.0470 |
|  |  | 0.7740 |
|  |  | 1.1450 |
|  |  | 1.1310 |
|  |  | 1.5950 |
|  |  | 0.9150 |
|  |  | 1.1310 |
|  |  | 0.7670 |
|  |  | 1.2980 |
|  |  | 0.8310 |
|  |  | 0.9630 |
|  |  | 1.0690 |
|  |  | 1.0690 |
|  |  | 1.3990 |
|  |  | 0.6810 |
|  |  | 1.0690 |
|  |  | 1.3330 |
|  |  | 1.0640 |
|  |  | 1.0660 |
|  |  | 1.2560 |
|  |  | 1.4460 |
|  |  | 0.9160 |
|  |  | 0.7320 |
|  |  | 1.4490 |
|  |  | 1.0900 |
|  |  | 0.7660 |
|  |  | 0.8550 |
|  |  | 1.1190 |
|  |  | 1.2560 |
|  |  | 0.8880 |
|  |  | 0.9040 |
|  |  | 0.5710 |
|  |  | 0.8030 |
|  |  | 0.9400 |
|  |  | 0.7270 |
|  |  | 1.2760 |
|  |  | 0.9700 |
|  |  | 0.7840 |
|  |  | 0.9040 |
|  |  | 0.8880 |
|  |  | 0.6860 |
|  |  | 1.3620 |
|  |  | 1.0640 |
|  |  | 1.1310 |
|  |  | 1.1700 |
|  |  | 1.0640 |
|  |  | 1.0530 |
|  |  | 1.1450 |
|  |  | 0.9570 |
|  |  | 1.0690 |
|  |  | 0.9630 |
|  |  | 1.1700 |
|  |  | 1.2030 |
|  |  | 0.9810 |
|  |  | 0.9570 |
|  |  | 0.9630 |
|  |  | 0.9570 |
|  |  | 0.8570 |
|  |  | 0.7740 |
|  |  | 0.8510 |
|  |  | 0.9090 |
|  |  | 1.2850 |
|  |  | 0.9630 |
|  |  | 0.9630 |
|  |  | 0.9810 |
|  |  | 0.9510 |
|  |  | 1.0090 |
|  |  | 1.0470 |
|  |  | 1.4600 |
|  |  | 1.4110 |
|  |  | 1.8420 |
|  |  | 1.0174 |
|  |  | 1.2140 |
|  |  | 2.0740 |
|  |  | 1.3900 |
|  |  | 0.9830 |
|  |  | 1.3070 |
|  |  | 1.4110 |
|  |  | 0.9270 |
|  |  | 1.8070 |
|  |  | 1.5080 |
|  |  | 1.1940 |
|  |  | 1.2140 |
|  |  | 0.7680 |
|  |  | 1.8740 |
|  |  | 1.5350 |
|  |  | 1.5350 |
|  |  | 1.1940 |
|  |  | 1.1990 |
|  |  | 1.0250 |
|  |  | 1.3120 |
|  |  | 1.6200 |
|  |  | 1.9350 |
|  |  | 1.0420 |
|  |  | 0.7860 |
|  |  | 1.1600 |
|  |  | 0.9900 |
|  |  | 1.0020 |
|  |  | 1.0570 |
|  |  | 1.4070 |
|  |  | 0.8340 |
|  |  | 1.6670 |
|  |  | 1.3730 |
|  |  | 1.2190 |
|  |  | 1.4250 |
|  |  | 1.7260 |
|  |  | 1.5050 |
|  |  | 1.9740 |
|  |  | 1.2230 |
|  |  | 1.3120 |
|  |  | 1.4420 |
|  |  | 1.1440 |
|  |  | 1.0780 |
|  |  | 1.0810 |
|  |  | 1.3270 |
|  |  | 1.7710 |
|  |  | 1.1830 |
|  |  | 1.6220 |
|  |  | 1.0420 |
|  |  | 1.5350 |
|  |  | 0.9350 |
|  |  | 1.7160 |
|  |  | 0.6210 |
|  |  | 0.9320 |
|  |  | 1.0580 |
|  |  | 1.1500 |
|  |  | 1.2520 |
|  |  | 1.1650 |
|  |  | 1.3180 |
|  |  | 0.9820 |
|  |  | 1.6040 |
|  |  | 1.4510 |
|  |  | 1.1890 |
|  |  | 0.7920 |
|  |  | 1.0630 |
|  |  | 1.1650 |
|  |  | 0.8790 |
|  |  | 1.3050 |
|  |  | 1.3980 |
|  |  | 1.7370 |
|  |  | 1.8710 |
|  |  | 1.3200 |
|  |  | 1.4590 |
|  |  | 1.0330 |
|  |  | 1.8180 |
|  |  | 1.7160 |
|  |  | 1.7280 |
|  |  | 1.4250 |
|  |  | 1.7030 |
|  |  | 2.0570 |
|  |  | 1.6920 |
|  |  | 1.2770 |
|  |  | 1.1810 |
|  |  | 1.8020 |
|  |  | 1.6820 |
|  |  | 1.9760 |
|  |  | 1.3110 |
|  |  | 1.9610 |
|  |  | 0.9730 |
|  |  | 1.2500 |
|  |  | 1.9870 |
|  |  | 2.2230 |
|  |  | 1.6040 |
|  |  | 1.0810 |
|  |  | 1.2320 |
|  |  | 0.7410 |
|  |  | 1.1990 |
|  |  | 1.7670 |
|  |  | 1.0000 |
|  |  | 1.3170 |
|  |  | 1.0400 |
|  |  | 1.3860 |
|  |  | 1.1170 |
|  |  | 0.8510 |
|  |  | 1.4980 |
|  |  | 1.2090 |
|  |  | 1.1690 |
|  |  | 1.6160 |
|  |  | 0.9820 |
|  |  | 1.2700 |
|  |  | 1.5130 |
|  |  | 1.5570 |
|  |  | 1.0740 |
|  |  | 1.0700 |
|  |  | 1.1940 |
|  |  | 1.0740 |
|  |  | 1.7540 |
|  |  | 1.1690 |
|  |  | 1.5400 |
|  |  | 1.0700 |
|  |  | 1.2710 |
|  |  | 1.1030 |
|  |  | 0.7640 |
|  |  | 0.6860 |
|  |  | 1.6520 |
|  |  | 1.4690 |
|  |  | 1.3720 |
|  |  | 1.3290 |
|  |  | 0.9700 |
|  |  | 1.5640 |
|  |  | 1.1940 |
|  |  | 1.0660 |
|  |  | 0.5450 |
|  |  | 1.0390 |
|  |  | 1.8660 |
|  |  | 1.2770 |
|  |  | 1.1910 |
|  |  | 1.1450 |
|  |  | 1.2990 |
|  |  | 0.7700 |
|  |  | 1.8410 |
|  |  | 1.5220 |
|  |  | 1.1570 |
|  |  | 1.6760 |
|  |  | 1.5830 |
|  |  | 1.8410 |
|  |  | 1.5080 |
|  |  | 1.8660 |
|  |  | 0.8550 |
|  |  | 0.6860 |
|  |  | 1.3640 |
|  |  | 2.1810 |
|  |  | 1.7100 |
|  |  | 1.6260 |
|  |  | 0.9000 |
|  |  | 1.2040 |
|  |  | 1.4470 |
|  |  | 1.3000 |
|  |  | 1.9840 |
|  |  | 1.6850 |
|  |  | 1.3220 |
|  |  | 1.5890 |
|  |  | 1.1550 |
|  |  | 1.2510 |
|  |  | 1.4570 |
|  |  | 0.8420 |
|  |  | 1.3090 |
|  |  | 1.2120 |
|  |  | 0.9160 |
|  |  | 1.4730 |
|  |  | 1.5300 |
|  |  | 1.7020 |
|  |  | 1.7700 |
|  |  | 1.3700 |
|  |  | 1.5520 |
|  |  | 1.3700 |
|  |  | 1.6180 |
|  |  | 1.3610 |
|  |  | 1.3430 |
|  |  | 1.8330 |
|  |  | 1.2930 |
|  |  | 0.8200 |
|  |  | 1.4610 |
|  |  | 0.5450 |
|  |  | 1.3680 |
|  |  | 0.6840 |
|  |  | 1.1750 |
|  |  | 1.6350 |
|  |  | 1.2950 |
|  |  | 0.9630 |
|  |  | 1.1460 |
|  |  | 1.5000 |
|  |  | 1.2370 |
|  |  | 1.4850 |
|  |  | 1.4850 |
|  |  | 1.4310 |
|  |  | 1.4720 |
|  |  | 1.5170 |
|  |  | 1.2130 |
|  |  | 1.1990 |
|  |  | 1.4310 |
|  |  | 1.8690 |
|  |  | 1.1200 |
|  |  | 1.0330 |
|  |  | 1.6470 |
|  |  | 1.2750 |
|  |  | 1.3340 |
|  |  | 1.2520 |
|  |  | 1.2750 |
|  |  | 1.2520 |
|  |  | 1.6730 |
|  |  | 1.3110 |
|  |  | 0.8590 |
|  |  | 1.2520 |
|  |  | 1.4740 |
|  |  | 1.2810 |
|  |  | 1.2980 |
|  |  | 1.1560 |
|  |  | 1.1440 |
|  |  | 0.8780 |
|  |  | 1.0570 |
|  |  | 1.1030 |
|  |  | 0.8520 |
|  |  | 1.2910 |
|  |  | 2.2110 |
|  |  | 1.1990 |
|  |  | 0.9270 |
|  |  | 0.7080 |
|  |  | 0.6650 |
|  |  | 1.4810 |
|  |  | 1.2090 |
|  |  | 0.8610 |
|  |  | 1.2180 |
|  |  | 0.8330 |
|  |  | 1.2420 |
|  |  | 1.0810 |
|  |  | 0.9270 |
|  |  | 1.2030 |
|  |  | 1.0890 |
|  |  | 1.3110 |
|  |  | 1.3780 |
|  |  | 0.9820 |
|  |  | 0.6580 |
|  |  | 1.2650 |
|  |  | 1.3450 |
|  |  | 1.1450 |
|  |  | 0.9190 |
|  |  | 0.7320 |
|  |  | 0.8030 |
|  |  | 0.7320 |
|  |  | 0.7840 |
|  |  | 0.9920 |
|  |  | 0.7700 |
|  |  | 0.8030 |
|  |  | 1.2180 |
|  |  | 0.8510 |
|  |  | 1.6430 |
|  |  | 0.6160 |
|  |  | 0.9160 |
|  |  | 0.9200 |
|  |  | 1.1570 |
|  |  | 1.1850 |
|  |  | 0.8760 |
|  |  | 1.0520 |
|  |  | 1.5320 |
|  |  | 1.0210 |
|  |  | 1.2030 |
|  |  | 0.9040 |
|  |  | 0.9400 |
|  |  | 0.9700 |
|  |  | 0.9000 |
|  |  | 1.2180 |
|  |  | 1.2650 |
|  |  | 1.2180 |
|  |  | 0.9040 |
|  |  | 1.1090 |
|  |  | 1.2650 |
|  |  | 1.2270 |
|  |  | 1.2180 |
|  |  | 1.8410 |
|  |  | 0.8380 |
|  |  | 0.9200 |
|  |  | 0.7020 |
|  |  | 0.7220 |
|  |  | 1.5640 |
|  |  | 1.2760 |
|  |  | 1.2180 |
|  |  | 1.4880 |
|  |  | 1.8890 |
|  |  | 0.6860 |
|  |  | 0.7840 |
|  |  | 1.3290 |
|  |  | 1.0350 |
|  |  | 1.1450 |
|  |  | 0.9700 |
|  |  | 1.2980 |
|  |  | 0.7520 |
|  |  | 1.0640 |
|  |  | 1.2850 |
|  |  | 1.1450 |
|  |  | 1.4270 |
|  |  | 1.3870 |
|  |  | 1.6090 |
|  |  | 1.8110 |
|  |  | 1.6610 |
|  |  | 0.7440 |
|  |  | 0.6730 |
|  |  | 0.9510 |
|  |  | 1.0640 |
|  |  | 0.9630 |
|  |  | 1.0030 |
|  |  | 0.5960 |
|  |  | 1.0390 |
|  |  | 1.5990 |
|  |  | 0.9510 |
|  |  | 1.4460 |
|  |  | 1.0830 |
|  |  | 0.7270 |
|  |  | 1.5220 |
|  |  | 1.4490 |

**Intensity of red collagen fibers**

| WT | MFS^-^ | MFS^+^ |
| --- | --- | --- |
| 141.478900 | 172.062800 | 177.311400 |
| 153.066100 | 177.299100 | 178.556000 |
| 157.199100 | 178.618500 | 195.989400 |
| 149.579700 | 177.384100 | 207.832600 |
| 141.271400 | 185.186700 | 212.802600 |
|  |  | 187.272700 |
|  |  | 192.572000 |
|  |  | 195.679300 |
|  |  | 193.421200 |
|  |  | 218.038700 |

**Percent of Interlaminar-fiber adherence**

| WT | MFS^-^ | MFS^+^ |
| --- | --- | --- |
| 100.000000 | 54.320990 | 38.461540 |
| 94.623660 | 46.534650 | 53.191490 |
| 95.000000 | 50.000000 | 60.000000 |
| 96.000000 | 57.500000 | 52.000000 |
| 99.000000 | 52.592590 | 50.980390 |
| 97.000000 |  | 43.877550 |
|  |  | 50.458720 |
|  |  | 46.153850 |
|  |  | 44.000000 |
|  |  | 57.142860 |
|  |  |  |
|  |  |  |

**Kyphosis Index**

| WT | MFS^-^ | MFS^+^ |
| --- | --- | --- |
| 5.38 | 4.04 | 3.570000 |
| 4.80 | 4.11 | 3.880000 |
| 4.39 | 4.34 | 3.380000 |
| 5.14 | 5.28 | 3.530000 |
| 4.96 | 4.66 | 3.560000 |
| 3.68 |  | 2.043553 |
| 4.50 |  | 2.140279 |
| 3.96 |  | 2.215647 |
| 3.94 |  | 2.292396 |
| 4.29 |  | 2.349585 |
| 4.07 |  | 2.383186 |
|  |  | 2.501499 |
|  |  | 2.714367 |

**Point Biserial Correlation**

| KI | Presence Aortic Disease |
| --- | --- |
| 2.043553 | 1 |
| 2.140279 | 1 |
| 2.215647 | 1 |
| 2.292396 | 1 |
| 2.349585 | 1 |
| 2.383186 | 1 |
| 2.501499 | 1 |
| 2.714367 | 1 |
| 3.288633 | 0 |
| 3.665794 | 0 |
| 3.750338 | 0 |
| 3.823184 | 0 |
| 3.848245 | 0 |
| 3.919962 | 0 |
| 3.935834 | 0 |
| 4.017011 | 0 |
| 4.305757 | 0 |
| 4.482648 | 0 |
| 4.543151 | 0 |
| 4.738544 | 0 |
| 4.814506 | 0 |

**Pearson Correlation between area of aortic lumen and KI**

| Aortic Lumen Area | KI |
| --- | --- |
| 2811.11600 | 2.043553 |
| 2965.76700 | 2.140279 |
| 3367.28900 | 2.215647 |
| 3754.99000 | 2.292396 |
| 6964.29900 | 2.349585 |
| 35671.30000 | 2.383186 |
| 55977.09000 | 2.501499 |
| 57956.48000 | 2.714367 |
| 39313.02000 | 3.288633 |
| 49695.47000 | 3.665794 |
| 43398.46000 | 3.750338 |
| 56085.46000 | 4.017011 |
| 63417.65000 | 4.482648 |
| 61203.35000 | 3.823184 |
| 75514.50000 | 3.848245 |
| 60712.96000 | 3.919961 |
| 75516.59000 | 3.935834 |
| 64607.03000 | 4.305757 |
| 57471.03000 | 4.543151 |
| 58991.27000 | 4.738544 |
| 60768.71000 | 4.814506 |

**Aortic blood flow**

| WT | MFS^-^ | MFS^+^ |
| --- | --- | --- |
| 1.28090 | 1.53820 | 1.18770 |
| 1.36140 | 1.73620 | 1.22330 |
| 1.37970 | 1.61690 | 1.26530 |
| 1.31720 | 1.69120 | 1.24550 |
| 1.39140 | 1.56400 | 1.25790 |
| 1.34612 | 1.62930 | 1.49240 |
| 1.31970 | 1.49240 | 1.39180 |
| 1.34130 | 1.39180 | 1.48370 |
| 1.28490 | 1.48370 | 1.41150 |
| 1.29720 | 1.41150 | 1.48100 |
| 1.26710 | 1.48100 | 1.45208 |
| 1.30204 |  | 1.32930 |
| 1.75810 |  | 1.27250 |
| 1.92720 |  | 1.30680 |
| 1.91460 |  | 1.33610 |
| 1.98470 |  | 1.33950 |
| 1.92490 |  | 1.31684 |
| 1.90190 |  | 1.63230 |
| 1.70600 |  | 1.70230 |
| 1.67560 |  | 1.48690 |
| 1.71800 |  | 1.85100 |
| 1.69160 |  | 1.68880 |
| 1.75200 |  | 1.67226 |
| 1.70864 |  | 1.26470 |
| 1.76440 |  | 1.31470 |
| 1.77290 |  | 1.34670 |
| 1.78910 |  | 1.33360 |
| 1.78840 |  | 1.34450 |
| 1.82890 |  | 1.32084 |
| 1.78874 |  | 0.93700 |
| 1.25560 |  | 0.97330 |
| 1.40670 |  | 0.91690 |
| 1.56650 |  | 0.95490 |
| 1.75340 |  | 1.05130 |
| 1.81990 |  | 0.96668 |
| 1.56042 |  | 0.79590 |
| 1.58480 |  | 0.75270 |
| 1.71860 |  | 0.77160 |
| 1.77480 |  | 0.73800 |
| 1.82140 |  | 0.78070 |
| 1.81330 |  | 0.76778 |
| 1.74258 |  | 0.57050 |
| 1.53250 |  | 0.55500 |
| 1.49930 |  | 0.55430 |
| 1.42750 |  | 0.55970 |
| 1.34770 |  | 0.57300 |
| 1.38680 |  | 0.56250 |
| 1.43876 |  |  |
| 1.16910 |  |  |
| 1.34970 |  |  |
| 1.34110 |  |  |
| 1.40050 |  |  |
| 1.32590 |  |  |
| 1.31726 |  |  |

**Relative heart frequency**

| WT | MFS^-^ | MFS^+^ |
| --- | --- | --- |
| 120. | 180. | 180. |
| 240. | 240. | 180. |
| 120. | 180. | 180. |
| 120. | 180. | 300. |
| 120. | 180. | 240. |
| 120. | 240. | 240. |
| 120. |  | 300. |
| 180. |  | 240. |
| 180. |  | 300. |
| 180. |  | 300. |
|  |  | 240. |
|  |  | 180. |
|  |  | 180. |
|  |  | 180. |
|  |  | 180. |
|  |  | 180. |
|  |  | 180. |
|  |  | 240. |
|  |  | 240. |
|  |  | 240. |

**Total heart area**

| WT | MFS^-^ | MFS^+^ |
| --- | --- | --- |
| 873.041700 | 697.876600 | 1039.140000 |
| 707.509700 | 684.009500 | 815.407400 |
| 726.995900 | 747.619400 | 732.261500 |
| 772.511400 | 748.603400 | 918.231300 |
| 789.667400 | 787.908800 | 925.260800 |
|  | 772.910300 | 719.082200 |
|  |  | 787.541000 |

**Right ventricle (RV) thickness**

| WT | MFS^-^ | MFS^+^ |
| --- | --- | --- |
| 1.958097 | 3.263141 | 3.402129 |
| 1.413097 | 2.393477 | 3.064697 |
| 1.887162 | 2.233311 | 4.494443 |
| 2.486152 | 2.479757 | 4.854996 |
| 1.771826 | 1.990682 | 3.446466 |
| 1.673230 | 2.479757 | 2.842536 |
| 1.579890 | 2.311940 | 4.063881 |
| 3.835193 | 3.059483 | 5.107605 |
| 2.477858 | 2.142019 | 3.979421 |
| 2.678474 | 1.952024 | 3.476943 |
| 1.635904 | 2.292536 | 3.372502 |
| 1.771826 | 1.990682 | 4.343033 |
| 1.566806 | 2.683344 | 4.267122 |
| 2.242350 | 2.316482 | 3.360272 |
| 2.785844 | 2.758674 | 3.045324 |
| 3.043633 | 2.409890 | 5.854986 |
| 1.931635 | 3.660168 | 2.599492 |
| 1.733654 | 2.755815 | 2.866874 |
| 1.471964 | 2.820844 | 5.022252 |
| 1.936957 | 2.391281 | 2.637816 |
| 1.639047 | 2.321016 | 5.056973 |
| 2.906984 | 2.897134 | 5.081339 |
| 1.816290 | 2.248556 | 3.845244 |
| 2.342258 | 2.724165 | 3.323311 |
| 1.936957 | 2.102394 | 3.585521 |
| 1.758706 | 2.691167 | 5.917935 |
| 2.091537 | 2.392379 | 3.630454 |
| 1.844407 | 2.042802 | 3.177647 |
| 2.487186 | 2.252059 | 5.356949 |
| 1.618509 | 2.275277 | 4.772675 |
| 2.019515 | 2.313076 | 3.665020 |
| 1.972114 | 2.491386 | 3.146724 |
| 1.790528 | 2.827358 | 2.963095 |
| 2.524914 | 2.342428 | 2.943928 |
| 1.629153 | 1.945281 | 3.149993 |
| 1.723218 | 1.818199 | 2.374988 |
| 2.524914 | 1.870911 | 3.604847 |
| 3.045709 | 2.659737 | 2.470577 |
| 1.769861 | 3.362568 | 3.630454 |
| 2.146920 | 2.181860 | 3.184119 |
| 2.538408 | 3.176837 | 3.444226 |
| 2.683344 | 1.721739 | 2.571625 |
| 2.031192 | 2.008696 | 3.449451 |
| 1.790528 | 3.642483 | 3.304675 |
| 2.393477 | 2.025281 | 3.422490 |
| 2.801212 | 2.630978 | 3.796756 |
| 2.266018 | 2.288917 | 1.893967 |
|  | 2.295652 | 1.938285 |
|  | 2.944802 | 1.838818 |
|  | 2.009976 | 2.209985 |
|  | 2.013813 | 2.054296 |
|  | 2.389031 | 1.668610 |
|  | 3.961923 | 2.535347 |
|  | 3.593407 | 1.764549 |
|  | 2.209985 | 2.313517 |
|  |  | 2.727031 |
|  |  | 1.838818 |
|  |  | 3.401373 |
|  |  | 1.679370 |
|  |  | 1.959411 |
|  |  | 2.745838 |
|  |  | 3.504220 |
|  |  | 2.927273 |
|  |  | 3.561759 |
|  |  | 3.358740 |
|  |  | 2.686149 |
|  |  | 3.630454 |
|  |  | 3.758610 |
|  |  | 3.567534 |
|  |  | 3.890484 |
|  |  | 3.144270 |
|  |  | 3.349534 |
|  |  | 3.272594 |
|  |  | 4.023152 |
|  |  | 2.801503 |
|  |  | 3.983231 |
|  |  | 3.027181 |
|  |  | 2.290004 |
|  |  | 3.610553 |
|  |  | 3.244164 |
|  |  | 3.435248 |
|  |  | 4.335917 |
|  |  | 3.897753 |
|  |  | 2.085377 |
|  |  | 3.307010 |
|  |  | 2.633911 |
|  |  | 3.766134 |
|  |  | 3.604847 |
|  |  | 3.570587 |
|  |  | 2.948831 |
|  |  | 3.338225 |

**Left ventricle (LV) thickness**

| WT | MFS^-^ | MFS^+^ |
| --- | --- | --- |
| 8.476153 | 8.480100 | 10.332680 |
| 8.038945 | 10.004970 | 10.047580 |
| 12.127310 | 6.965718 | 14.277710 |
| 12.137280 | 10.390540 | 12.127310 |
| 9.895061 | 7.125325 | 11.968400 |
| 8.071210 | 8.779474 | 14.745840 |
| 12.081180 | 11.185090 | 12.647670 |
| 11.794050 | 9.258796 | 14.284920 |
| 8.560736 | 7.467075 | 12.208310 |
| 13.407540 | 7.783944 | 8.440254 |
| 13.007750 | 10.193270 | 8.981441 |
| 8.775957 | 6.888451 | 10.138590 |
| 6.161218 | 6.852871 | 13.030680 |
| 12.965940 | 6.905241 | 10.230810 |
| 10.126400 | 8.627805 | 14.168250 |
| 8.439949 | 8.613775 | 10.188470 |
| 8.015544 | 10.919440 | 16.220820 |
| 8.218437 | 10.117510 | 11.059230 |
| 7.828774 | 9.917171 | 9.482871 |
| 9.326087 | 6.102194 | 14.792360 |
| 8.544489 | 5.593975 | 11.186010 |
| 8.514017 | 11.671910 | 10.988270 |
| 9.105219 | 7.382535 | 11.959800 |
| 12.840300 | 7.232578 | 14.778960 |
| 7.713208 | 3.691623 | 11.101040 |
| 11.263270 | 5.363260 | 9.733549 |
| 10.350340 |  | 11.782050 |
| 10.946980 | 6.141685 | 15.869270 |
| 8.470384 | 7.425127 | 15.444590 |
| 10.760870 | 6.453804 | 15.154460 |
| 6.903035 | 6.952471 | 13.606250 |
| 7.661723 | 6.385031 | 11.257560 |
| 8.368541 | 7.251084 | 7.579266 |
| 8.602662 | 4.495358 | 15.679550 |
| 5.159479 | 4.930285 | 11.625280 |
| 5.992690 |  | 12.792320 |
| 5.929208 | 6.310931 | 13.376030 |
| 6.327565 | 6.377206 | 11.411030 |
| 9.232119 | 5.577980 | 9.571194 |
| 9.658080 | 7.425127 | 9.672819 |
|  | 6.145535 | 8.065788 |
| 8.308979 | 7.549721 | 12.537940 |
| 8.417072 | 6.398188 | 11.745080 |
| 5.234316 | 7.859754 | 9.719527 |
| 5.574681 |  | 11.702940 |
| 5.576567 | 7.227126 | 10.330680 |
| 5.907896 | 6.622612 | 8.257794 |
|  | 5.960593 | 9.476627 |
| 6.640048 | 6.745667 | 11.854550 |
| 8.578496 | 6.243531 | 11.572260 |
| 7.393206 | 7.423003 | 12.987950 |
| 5.708828 | 7.542756 | 13.561730 |
| 7.556679 | 8.585233 | 11.517870 |
| 7.748974 |  | 11.832610 |
|  | 11.168200 | 12.837100 |
|  | 11.750000 | 16.332120 |
|  | 8.559483 | 11.780300 |
|  | 8.050033 | 12.032510 |
|  | 7.008563 | 13.041930 |
|  | 9.774379 | 12.273260 |
|  |  | 13.113940 |
|  | 12.213210 | 12.676730 |
|  | 6.828525 | 16.895290 |
|  | 10.006860 | 10.563450 |
|  | 9.934381 | 6.404099 |
|  | 8.119914 | 14.329520 |
|  | 6.216958 | 10.846850 |
|  |  | 9.201924 |
|  |  | 11.043630 |
|  |  | 12.218630 |
|  |  | 14.330060 |
|  |  | 11.320470 |
|  |  | 11.651150 |
|  |  | 11.048760 |
|  |  | 6.915667 |
|  |  | 10.607930 |
|  |  | 10.782850 |
|  |  | 9.778914 |
|  |  | 12.835690 |
|  |  | 13.362940 |
|  |  | 10.608180 |
|  |  | 12.914040 |
|  |  | 13.130020 |
|  |  | 11.725120 |
|  |  | 9.942536 |
|  |  | 9.870846 |
|  |  | 9.641910 |
|  |  | 10.466050 |
|  |  | 12.726970 |
|  |  | 11.243840 |
|  |  | 9.782861 |
|  |  | 8.147367 |
|  |  | 12.150200 |
|  |  | 11.372180 |
|  |  | 8.790898 |
|  |  | 10.988560 |

**RV Lumen / Total Heart Area**

| WT | MFS^-^ | MFS^+^ |
| --- | --- | --- |
| 11.806590 | 7.034101 | 9.001938 |
| 11.257950 | 7.210633 | 2.292642 |
| 7.603558 | 12.090770 | 5.143697 |
| 8.588415 | 12.206610 | 3.959869 |
| 8.633712 | 7.348797 | 6.085743 |
|  | 7.669720 | 3.926697 |
|  |  | 7.656969 |

**LV Lumen / Total Heart Area**

| WT | MFS- | MFS+ |
| --- | --- | --- |
| 11.217060 | 11.381910 | 2.203274 |
| 9.232671 | 11.898000 | 0.6882157 |
| 11.607090 | 8.493970 | 3.099182 |
| 9.687388 | 8.578221 | 7.437670 |
| 11.467450 | 3.598490 | 1.317612 |
|  | 2.795140 | 4.772133 |
|  |  | 4.576793 |
