# Supplementary material for: Association of thoracic spine deformity and cardiovascular disease in a mouse model for Marfan syndrome
Source: PLoS One. 2019 Nov 14;14(11):e0224581. doi: 10.1371/journal.pone.0224581 (PMC6855660; doi:10.1371/journal.pone.0224581)
Supplement: Attachment — Submitted filename: ResponseReviewer3.docx [file pone.0224581.s003.docx]

Reviewer #3: This manuscript presents the association of the thoracic kyphosis and the incidence of descending aortic aneurysm/dissection, using their kyphosis-prone Marfan mice (mgΔloxPneo: in-frame deletion of FBN1 exon 19-24). As authors mentioned, risk factors for type B aortic dissection remain elusive (line 297), and thus this study might provide practical information for prognostic stratification of patients with Marfan syndrome (MFS), however, data presentation is insufficient for drawing scientific conclusions. I have serious concerns on several issues in this paper.

(1) I was confused with the word of “vascular disease (defined at line 142)” used for “the presence of thoracic aneurysm and/or dissection”. It is better to use the word of “descending thoracic aortic aneurysm and/or dissection (dTAAD)” or something, because the mgΔloxPneo mice seem to have significant histological changes in entire aorta.

Thank you for the suggestion, we changed the nomenclature to dTAAD.

(2) Authors should show the objective definition and data for the “presence of descending aortic aneurysm (line 142)”. It might be difficult to evaluate due to the “dysmorphism of the aorta” in kyphosis mice, however, authors must show/compare the aortic lumen area when dividing into two groups. Fig. 1a, c are inappropriate images showing the presence of aneurysm.

The presence or absence of descending aortic aneurysm was defined by the histologic aspect and confirmed by two different pathologists. In this study, we found the saccular aneurysm, which is defined as a degenerative process in the aorta wall in bleb form (Kummar et al. 2010). We thank you for the suggestion and we changed the picture for better visualization of the aneurysm.

In addition, we included the measurement of the area of the aortic lumen in the three groups (WT, MFS+, and MFS-), in Figure 1. We observed a significant decreased of aorta's lumen in MFS+ when compared to WT and MFS- animals.

(3) Authors show the positive correlation between the presence of dTAAD (categorical) and kyphosis index (KI) (continuous), however, I think authors should show the correlation between the lumen area and KI, as I described above. Hopefully, it would be preferable to present the time serial data (e.g. at 3 months), because the mgΔloxPneo mice possibly develop kyphosis at more earlier ages than 6 months.

Thank you for the suggestion. We used Pearson's Correlation and observed a positive correlation between KI and area of the aorta's lumen (cor 0,7984, p<0,0001). Thus, the larger the spine tortuosity (lower KI), the lower the lumen area. We added this to the text and figure 3D.

Although the suggestion of the time serial analysis is very interesting, the focus of the present study was to characterize the cardiovascular phenotypes at an age where most of the animals showed some alteration, i.e., at 6 months (Lima et al., 2010). From that analysis we found the variability in dTAAD occurrence and went on to try to understand its origin.

(4) Ref 19 (Int J Cardiol 2015;194:7-12) shows the relation between vase tortuosity and aortic disease in patients with MFS (line 314), however, “aortic root dilatation” is defined as aortic disease in Ref 19. Authors should rather show the data of aortic root diameter as a control as requested by reviewer #1. It is important to examine the influence of the kyphosis on the location of aortic aneurysm and dissection.

Indeed, in this work we focused in the descending aorta due to the frequent finding of dissection and aneurysms in descending thoracic aorta (type B) in MFS patients. Please note that in reference 19, in addition to correlating aortic tortuosity to aortic root diameter, the authors also showed that those patients with higher aortic tortuosity had a higher probability of developing aortic dissection (“After 49.3 ± 8.8 months follow-up, 33 patients met the combined clinical endpoint (7 dissections) with a significantly higher ATI at baseline than patients without endpoint (1.98 ± 0.2 vs. 1.86 ± 0.2, p=0.002). Patients with an ATI>1.95 had a 12.8 times higher probability of meeting the combined endpoint (log rank-test, p<0.001) and a 12.1 times higher probability of developing an aortic dissection (log rank-test, p=0.003) compared to patients with an ATI<1.95. “).

They go on to suggest that quantification of aortic tortuosity may improve prediction of type B aortic dissection. We were referring to that aortic phenotype (dissection), not to the increase in aortic root diameter, and thus we have changed our text accordingly to make this point more clear.

(5) Authors show the hemodynamic data obtained by aortic blood blow and cardiac histological analyses. However, it seems inaccurate for drawing any conclusions in the present methods; heart rates were very slow and cardiac index should be obtained by echocardiography. Especially, it is very important to analyze the cardiac phenotypes of mgΔloxPneo mice by echocardiography and/or transcriptome analysis. Previously reported clinical and experimental Marfan hearts develop dilated cardiomyopathy (DCM)-like phenotypes after cardiac loading, but not concentric hypertrophy in this study. It’s possible, but should be evaluated by accurate modalities in the future. I do not think, hemodynamic data is necessarily included in this study, mainly showing the association of the thoracic kyphosis and the incidence of descending aortic aneurysm/dissection.

Thank you for the careful analysis of our data. We chose to include the hemodynamic data in order to support our hypothesis of disturbed blood flow in the animals with dTAAD/low KI. The analysis of the heart was a suggestion from one of the reviewers based on our suggestion of high-output cardiac failure, and revealed another important phenotype of this MFS model.

The low values of the heart rates are probably due to the anesthetic procedure. However, although the heart rates were slow, all animals were submitted to the same procedure, thus, the differences found are significant.

Indeed DCM-like phenotypes are present in some MFS patients and in the hypomorphic model mgR (Cook et al., 2014). Nevertheless, LV hypertrophy has also been described in MFS patients and in a different mouse model for the disease (Tae et al. 2016; Arumamate et al. 2018). We agree that echocardiography and/or trancriptome analysis of the heart will provide more insights on the cardiac phenotype, and we should perform them in a future study that will focus on the cardiac phenotypes of our MFS model. In this study we presented those data to corroborate our hypothesis of high-output cardiac failure. Nevertheless, despite not being our initial goal, our histological description revealed an important cardiac phenotype that deserves further investigation. We have modified the text in order to make these points more clear.

- Cook JR, Carta L, Bénard L, Chemaly ER, Chiu E, Rao SK, Hampton TG, Yurchenco P; GenTAC Registry Consortium, Costa KD, Hajjar RJ, Ramirez F. Abnormal muscle mechanosignaling triggers cardiomyopathy in mice with Marfan syndrome. J Clin Invest. 2014;124(3):1329-39.
- [Tae HJ](https://www.ncbi.nlm.nih.gov/pubmed/?term=Tae%20HJ%5BAuthor%5D&cauthor=true&cauthor_uid=26566724), [Petrashevskaya N](https://www.ncbi.nlm.nih.gov/pubmed/?term=Petrashevskaya%20N%5BAuthor%5D&cauthor=true&cauthor_uid=26566724), [Marshall S](https://www.ncbi.nlm.nih.gov/pubmed/?term=Marshall%20S%5BAuthor%5D&cauthor=true&cauthor_uid=26566724), [Krawczyk M](https://www.ncbi.nlm.nih.gov/pubmed/?term=Krawczyk%20M%5BAuthor%5D&cauthor=true&cauthor_uid=26566724), [Talan M](https://www.ncbi.nlm.nih.gov/pubmed/?term=Talan%20M%5BAuthor%5D&cauthor=true&cauthor_uid=26566724). Cardiac remodeling in the mouse model of Marfan syndrome develops into two distinctive phenotypes. [Am J Physiol Heart Circ Physiol.](https://www.ncbi.nlm.nih.gov/pubmed/?term=Cardiac+remodeling+in+the+mouse+model+of+Marfan+syndrome+develops+into+two+distinctive+phenotype) 2016; 310(2):H290-9.
- [Arunamata AA](https://www.ncbi.nlm.nih.gov/pubmed/?term=Arunamata%20AA%5BAuthor%5D&cauthor=true&cauthor_uid=29972109), [Nguyen CT](https://www.ncbi.nlm.nih.gov/pubmed/?term=Nguyen%20CT%5BAuthor%5D&cauthor=true&cauthor_uid=29972109), [Ceresnak SR](https://www.ncbi.nlm.nih.gov/pubmed/?term=Ceresnak%20SR%5BAuthor%5D&cauthor=true&cauthor_uid=29972109), [Dubin AM](https://www.ncbi.nlm.nih.gov/pubmed/?term=Dubin%20AM%5BAuthor%5D&cauthor=true&cauthor_uid=29972109), [Olson IL](https://www.ncbi.nlm.nih.gov/pubmed/?term=Olson%20IL%5BAuthor%5D&cauthor=true&cauthor_uid=29972109), [Murphy DJ](https://www.ncbi.nlm.nih.gov/pubmed/?term=Murphy%20DJ%5BAuthor%5D&cauthor=true&cauthor_uid=29972109), [Selamet Tierney, ES](https://www.ncbi.nlm.nih.gov/pubmed/?term=Selamet%20Tierney%20ES%5BAuthor%5D&cauthor=true&cauthor_uid=29972109). Utility of serial 12-lead electrocardiograms in children with Marfan syndrome. [Cardiol Young.](https://www.ncbi.nlm.nih.gov/pubmed/?term=Utility+of+serial+12-lead+electrocardiograms+in+children+with+Marfan+syndrome) 2018;28(8):1009-1013.

Minor points;

(6) It is better to explain more mgΔloxPneo mice. (e.g. in-frame deletion of FBN1 exon 19-24; susceptibility of the kyphosis).

Thank you for the suggestion, we included more info about the mice.

(7) Line 143, 151; aneurism

We apologize, we have corrected the text.

(8) Comma in the numbers in the part of method.

We apologize, we have corrected the text.
